# Detection of SARS-CoV-2 viral proteins and genomic sequences in human brainstem nuclei

**DOI:** 10.1038/s41531-023-00467-3

**Published:** 2023-02-13

**Authors:** Aron Emmi, Stefania Rizzo, Luisa Barzon, Michele Sandre, Elisa Carturan, Alessandro Sinigaglia, Silvia Riccetti, Mila Della Barbera, Rafael Boscolo-Berto, Patrizia Cocco, Veronica Macchi, Angelo Antonini, Monica De Gaspari, Cristina Basso, Raffaele De Caro, Andrea Porzionato

**Affiliations:** 1 Institute of Human Anatomy, Department of Neuroscience, University of Padova, Padova, Italy; 2 Center for Neurodegenerative Disease Research (CESNE), University of Padova, Padova, Italy; 3 Department of Cardio-Thoracic-Vascular Sciences & Public Health, University of Padova, Padova, Italy; 4 Department of Molecular Medicine, University of Padova, Padova, Italy; 5 Department of Neuroscience, University of Padova, Padova, Italy; 6 Pathology and Histopathology Unit, Ospedali Riuniti Padova Sud, Padova, Italy; 7 Movement Disorders Unit, Department of Neuroscience, University of Padova, Padova, Italy

**Keywords:** Neuroimmunology, Neurodegenerative diseases, Brain, Chronic inflammation

## Abstract

Neurological manifestations are common in COVID-19, the disease caused by SARS-CoV-2. Despite reports of SARS-CoV-2 detection in the brain and cerebrospinal fluid of COVID-19 patients, it is still unclear whether the virus can infect the central nervous system, and which neuropathological alterations can be ascribed to viral tropism, rather than immune-mediated mechanisms. Here, we assess neuropathological alterations in 24 COVID-19 patients and 18 matched controls who died due to pneumonia/respiratory failure. Aside from a wide spectrum of neuropathological alterations, SARS-CoV-2-immunoreactive neurons were detected in the dorsal medulla and in the substantia nigra of five COVID-19 subjects. Viral RNA was also detected by real-time RT-PCR. Quantification of reactive microglia revealed an anatomically segregated pattern of inflammation within affected brainstem regions, and was higher when compared to controls. While the results of this study support the neuroinvasive potential of SARS-CoV-2 and characterize the role of brainstem inflammation in COVID-19, its potential implications for neurodegeneration, especially in Parkinson’s disease, require further investigations.

## Introduction

Neurological manifestations are common in coronavirus disease 19 (COVID-19), the disease caused by severe acute respiratory syndrome coronavirus-2 (SARS-CoV-2)^1–5^. Symptoms range from anosmia, ageusia, dizziness and headache, which are commonly reported by patients with mild disease, to altered mental status, neuropsychiatric disorders, stroke, and rarely, meningitis, encephalitis, and polyneuritis, which occur in hospitalized patients with severe disease^1,5^. Between 10 to 30% of COVID-19 patients experience long-term sequelae, referred to as “long COVID”, including neurological manifestations such as hyposmia, hypogeusia, headaches, fatigue, sleep disorders, pain, and cognitive impairment^3^. Despite reports of SARS-CoV-2 detection in the brain and cerebrospinal fluid of COVID-19 patients^2,3,6^, it is still unclear whether the virus can infect the central nervous system (CNS), and which neuropathological alterations can occur following infection. In particular, it remains to be elucidated whether neuropathological and neurological manifestations encountered in COVID-19 are a direct consequence of viral invasion, are due to post-infectious immune-mediated disease, or are the result of systemic disease^1,6,7^. Studies on human neural cell cultures and brain organoids report conflicting data on SARS-CoV-2 neurotropism^8^. Overall, they suggest that SARS-CoV-2 does not infect and replicate efficiently in human neural cells, while it can replicate at high rates in choroid plexus epithelial cells^9–11^. At variance, intranasal inoculation of SARS-CoV-2 in transgenic mice overexpressing human ACE2 under the K18 promoter resulted in brain invasion and widespread infection of neurons, radial glia and neuronal progenitor cells^12,13^, while also being supported by the notion that other coronaviruses, such as SARS-CoV and MERS-CoV, are able to infect the CNS in both humans and animal models^14^.

Data deriving from large autopsy studies on COVID-19 decedents support the neuroinvasive potential of SARS-CoV-2^14–17^, even though infection was mostly limited to sparse cells in the brainstem, hypothalamus and cerebellum, and was not associated to encephalitis or other virus-specific changes^16,17^. Conversely, other studies did not detect SARS-CoV-2 antigens or genomic sequences in the brain^7,14,18–20^. In numerous instances, neuropathological changes in COVID-19 were moderate and represented predominantly by ischemic lesions, astrogliosis, microglial nodules, and cytotoxic T lymphocyte infiltrates, most pronounced in the brainstem, cerebellum, and meninges^7,15,16,18,21^. In most studies, no direct link between encountered neuropathological alterations and direct viral invasion could be established, with systemic inflammation and hypoxia playing a likely major role in mediating brain immune response^19^. Single-nucleus gene-expression profiling of frontal cortex and choroid plexus tissues from severe COVID-19 patients showed broad perturbations, with upregulation of genes involved in innate antiviral response and inflammation, microglial activation and neurodegeneration^22^, but no direct evidence of viral tropism was found; similarly, Fullard et al.^20^ did not detect viral transcripts and S proteins in different brain regions of COVID-19 subjects. On the other hand, deep spatial profiling of the local immune response in COVID-19 brains through imaging mass spectrometry revealed significant immune activation in the CNS, with pronounced neuropathological changes (such as astrogliosis, axonal damage, and blood-brain-barrier leakage), and detected viral antigens in ACE2-positive cells in the perivascular compartment^19^. Recently, Stein et al.^17^ confirmed SARS-CoV-2 neurotropism in a large autopsy case series, also demonstrating that viral replication can occur in numerous tissues, including the brain, and can persist for months following symptom onset. SARS-CoV-2 genomic sequences and viral proteins were detected in the spinal ganglia, cerebellum and hypothalamus, but were not associated to inflammation or neuropathological changes. Hence, SARS-CoV-2 viral neurotropism has been documented only in a subset of cases to date, and was not consistenly reproduced throughout available studies, while widespread neuropathological sequelae (such as astrogliosis, microgliosis, lymphocyte infiltration, microvascular injury, fibrinogen leakage) have been documented in most examined specimens. The possibility and extent of direct viral invasion, and eventual associated long-term sequelae of infection, remain to be investigated. This appears to be particularly concerning for elderly subjects, with known susceptibility for COVID-19, and for patients vulnerable to—or already suffering from—neurodegenerative diseases, such as Parkinson’s Disease. Indeed, the hypothesis that viral infections, such as COVID-19, may trigger or precipitate neurological manifestations and neurodegeneration, either through direct invasion or indirectly via neuroinflammation, requires particular attention in future post-pandemic scenarios. As the clinical burden of post-infectious syndromes, i.e. long COVID, appears to be continuously increasing, particular care must be taken to investigate yet unknown factors underlying potentially severe long-term sequelae in COVID-19.

In the present study, we assess the neuropathological changes of 24 patients who died following a diagnosis of SARS-CoV-2 infection in Italy during the COVID-19 pandemic (from March 2020 to May 2021) and 18 age-matched controls with comparable medical conditions who died due to pneumonia and/or respiratory failure.

## Results

### The main cause of death in COVID-19 subjects was diffuse alveolar damage

**Fig. 1:**
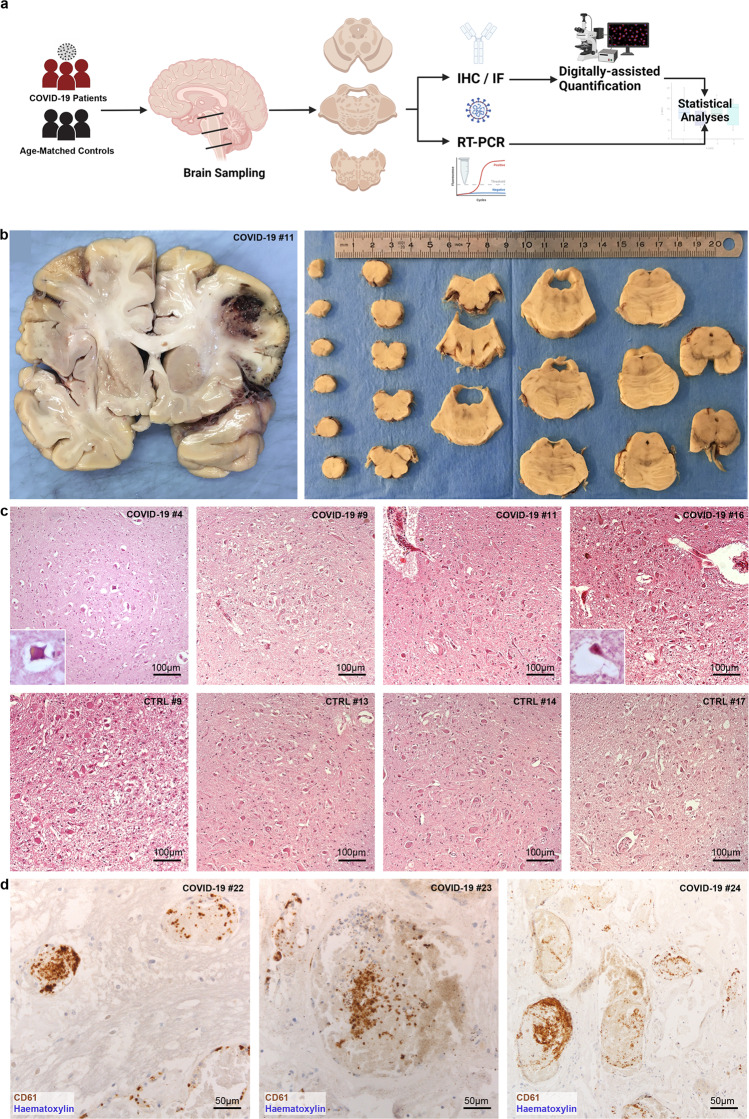
Study workflow and histopathological findings. **a** Study worflow: brain sections of multiple sites, with particular regard to the brainstem, were sampled from 24 COVID-19 patients and 18 age- and sex-matched controls who died due to pneumonia and/or respiratory failure. **b** Left, coronal brain section of Subject #11 revealing extensive hemorrhagic injury in the territory of the middle cerebral artery. Right, sampling procedure of the brainstem through axial sections passing perpendicularly to the floor of the fourth ventricle. **c** Haematoxylin and eosin photomicrographs of the Dorsal Motor Nucleus of the Vagus in the medulla oblongata displaying various degrees of hypoxic/ischemic damage in COVID-19 subjects (upper row) and controls (lower row). **d** Platelet microthrombi at the level of the pons and cerebral cortex, CD61 immunohistochemistry.

Twenty-four COVID-19 patients were included in our study (Fig. 1a). In all patients, SARS-CoV-2 RNA was detected by molecular testing in rhino-pharyngeal swabs. Eleven were females, while 13 were males. The mean age of the included subjects was 73 ± 13.7 years. Most included subjects were affected by preexisting chronic medical conditions. Eleven patients (7 female, 4 male) were affected by neurological or neurodegenerative disease prior to SARS-CoV-2 infection. Twenty-three patients were hospitalized prior to death. Patients were hospitalized for 14.5 ± 11.3 days and died 1 to 38 days following admission. Eleven subjects were admitted to the ICU during hospitalization and received intensive oxygen therapy (IOT) (i.e., the administration of supplemental oxygen via nasal cannulae, face masks, or tracheal intubation). Fifteen subjects received antithrombotic therapy during hospitalization and were treated with corticosteroid medication. The available clinical data for our cohort is reported in Table 1.

**Table 1:** Clinical data of the COVID-19 group.

ID	Age	Sex	Hospitalization (days)	ICU	Intensive oxygen therapy	Anti-thrombotic medication	Steroid medication	Hypertension	PMI	Antemortem head CT	Neurological signs	Neuropathological evaluation	Brainstem hypoxic damage	Microthrombosis (NON-CNS)	Cause of death
#1	87	F	2	N	N	Y	Y	Y	4	NA	Cognitive decline	AD neuropathological changes, CAA	Mild	Lungs, liver	Diffuse alveolar damage
#2	92	F	5	N	N	N	Y	N	1	NA	Cognitive decline, Alzheimer type	AD neuropathological changes, CAA	Mild	Lungs	Diffuse aveolar damage, intestinal infarction
#3	83	M	8	N	N	Y	Y	Y	2	Cerebral and cerebellar atrophy, chronic ischemic vascular disease	Frontoparietal ischemic insult (old) episodes of hepatic encephalopathy (HCV+)	Multi-infarct dementia, arteriosclerosis	Moderate	No	Diffuse alveolar damage, hepatic cyrrhosis
#4	97	F	9	N	N	Y	Y	Y	5	NA	Vascular dementia	Vascular dementia, arterioscelrosis, diffuse hypoxic/ischemic damage	Mild	Lungs	Diffuse alveolar damage, cardiac amyloidosis
#5	78	F	15	N	N	Y	Y	Y	5	NA	NA	Early AD neuropathological changes, diffuse hypoxic/ischemic damage	Mild	Liver	Pneumonia, aspergillus bronchopneumonia
#6	74	M	13	Y	Y	Y	Y	Y	4	Vascular calcification, expansive lesion of the right frontal lobe and right cerebellar hemisphere in patient with pulmonary neoplasia	NA	Small cell metastatic lung carcinoma with right cerebellar and frontal metastases	Mild	No	Small cell metastatic lung carcinoma, diffuse alveolar damage
#7	58	F	11	Y	Y	Y	N	N	2	Extensive ischemic lesion of the territory of the right PCA, occulsion of the PCA	NA	Medial temporal lobe infarction	Moderate	No	Acute myocardial infarction, cardiogenic shock
#8	50	M	1	N	N	N	N	N	3	NA	NA	Arteriosclerosis, diffuse hypoxic/ischemic damage	No detectable changes	No	Coronary atherosclerosis and myocardiosclerosis
#9	81	F	30	N	N	N	N	Y	3	Ischemic regions in the MCA territory and diffuse cerebral and cerebellar atrophy due to chronic ischemic vascular disease	Soporous status	Mixed dementia with AD neuropathological changes, CAA and chronic ischemic vascular disease	Moderate	Lungs, liver	Atherosclerotic aortic aneurysm, pneumonia
#10	60	M	30	Y	Y	Y	Y	N	3	NA	NA	Diffuse hypoxic/ischemic damage	Mild	Heart	Pneumonia with emphysema
#11	55	M	15	Y	Y	Y	Y	N	2	NA	NA	CNS microthromboses, haemorrhagic injury in the territory of the right MCA	Moderate	No	Pulmonary thromboembolism with infarcts
#12	62	M	27	Y	Y	Y	Y	N	2	NA	NA	Arteriosclerosis, diffuse hypoxic/ischemic damage	Mild	Lungs	Pneumonia with hemorrhages. Intestinal and hepatic infarcts
#13	73	M	21	Y	Y	Y	Y	Y	2	NA	NA	Diffuse hypoxic/ischemic damage	Moderate	No	Pneumothorax, Pneumonia, right pleurodesis,
#14	58	F	24	Y	Y	Y	Y	Y	1	Right anisocoria	No relevant signs	Diffuse hypoxic/ischemic damage	Mild	Lungs	Pneumonia. Necrotic-haemorrhagic pancreatitis. Multiorgan failure
#15	49	F	3	Y	Y	Y	N	N	2	Confusion and hallucinations; CSF Streptococcus Pneumoniae +	NA	Acute purulent meningitis, post-anoxic pathology	Moderate	No	Acute purulent meningitis. Post-anoxic cerebral death
#16	72	M	10	Y	Y	Y	Y	Y	4	Hypostenia, dizziness, anosmia. Sudden fall	NA	Diffuse hypoxic/ischemic damage	Severe	No	Consolidative pneumonia. Hypertensive heart disease
#17	72	M	38	N	N	N	Y	N	1	NA	NA	CNS microthromboses, extensive haemorrhagic injury of the right cerebellar hemisphere, ischemic vascular disease	Mild	No	Multivascular obstructive coronary atherosclerosis. Left pulmonary infarct
#18	82	M	6	N	N	N	Y	Y	3	Acute neurological event: Anisocoria, non responding	NA	CNS microthromboses, ischemic vascular disease	Mild	No	Lobar pneumonia
#19	40	F	1	N	N	NA	NA	Y	ND	NA	NA	CNS microthromboses, diffuse hypoxic/ischemic damage	Moderate (medulla not sampled)	NA	Diffuse alveolar damage
#20	68	M	NA	N	N	NA	NA	Y	ND	NA	NA	CNS microthromboses, diffuse hypoxic/ischemic damage	Severe (medulla not sampled)	NA	Diffuse alveolar damage
#21	73	M	5	Y	Y	Y	Y	N	5	NA	NA	CNS microthromboses, cortical and subcortical haemorrages, global ischemia	Moderate	Lungs	Diffuse alveolar damage, Platelet/fibrin microthrombosis
#22	77	F	34	N	N	N	N	Y	5	No signs	NA	CNS microthromboses, ischemic vascular disease	Moderate to severe	Lungs	Chronic emphysema, diffuse alveolar damage and platelet/fibrin microthromboses
#23	84	M	12	Y	Y	Y	Y	Y	4	Chronic ischemic vascular disease	Cognitive decline	AD neuropathological changes, Parkinson’s Disease, CNS microthromboses, ischemic vascular disease	Moderate	Lungs	Chronic emphysema, bacterial pneumonia, diffuse alveolar damage lung platelet/fibrin microthrombosis
#24	89	F	1	N	N	N	N	Y	5	Chronic ischemic vascular disease, territorial ischemic injury (right occipital lobe, caudate nucleus and cerebellum)	Cognitive decline	AD neuropathological changes, CNS microthromboses, diffuse hypoxic/ischemic damage	Moderate	Lungs	Chronic emphysema, diffuse alveolar damage and platelet/fibrin microthromboses

### The main cause of death of the control cohort was respiratory failure and pneumonia, aside from other relevant comorbidities

Eighteen age- and sex-matched subjects with comparable ante-mortem medical conditions were included as controls. All patients were negative for SARS-CoV-2 infection or died prior to the COVID-19 pandemic in Italy. Eight were female, while ten were male. The mean age of included controls was 72 ± 12 years. The mean hospitalization time was 20 ± 15.6 days. Thirteen patients died due to pneumonia, while the remaining subjects died due to respiratory insufficiency, multiorgan failure or ischemic heart disease. One patient died due to septic shock. Five patients had a clinical diagnosis of cognitive decline. The available clinical data for the control group are reported in Table 2.

**Table 2:** Clinical data of the control group.

ID	Age	Sex	Hospitalization (days)	Hypertension	PMI	Antemortem head CT	Neurological signs	Neuropathological evaluation	Brainstem hypoxic damage	Microthrombosis (NON-CNS)	Cause of death
#1	83	M	10	Y	5	Cerebral atrophy, Chronic ischemic vascular disease	Cognitive decline	Mixed dementia with AD neuropathological changes ad chronic ischemic vascular disease	Moderate	No	Pneumonia, respiratory insufficiency, ischemic heart disease
#2	74	M	2	Y	4	Vascular calcification, ischemic heart disease	NA	Chronic ischemic vascular disease	Mild	No	Ischemic heart disease
#3	40	M	1	N	4	No signs	No signs	No detectable microscopical changes	No detectable microscopical changes	No	Haemorrhagic Shock
#4	79	F	31	Y	3	Cerebral atrophy, Chronic ischemic vascular disease	Cognitive decline, Alzheimer type	AD neuropathological changes, CAA, ischemic vascular disease	Mild	No	Pentalobar pneumonia, respiratory insufficiency
#5	62	M	22	Y	5	NA	NA	Diffuse hypoxic/ischemic damage	Mild	No	Pneumonia, respiratory insufficiency
#6	76	M	15	Y	6	NA	NA	Ischemic vascular disease, diffuse hypoxic/ischemic damage	Mild	No	Pneumonia, chronic ischemic vascular disease
#7	75	M	12	Y	4	NA	NA	Diffuse hypoxic/ischemic damage	Mild	No	Pneumonia, ischemic heart disease
#8	78	F	8	Y	3	No	NA	Diffuse hypoxic/ischemic damage	Mild	No	Pneumonia, ischemic heart disease
#9	71	F	40	Y	3	NA	NA	Diffuse hypoxic/ischemic damage	Moderate to severe	No	Acute respiratory failure, septic shock, peritonitis
#10	46	F	15	N	4	No signs	No signs	Diffuse hypoxic/ischemic damage	No detectable microscopical changes	No	Respiratory insufficiency, multiorgan failure, cervical neoplasia
#11	75	F	20	Y	5	Chronic Ischemic Vascular disease, Cerebral atrophy	NA	Vascular dementia, ischemic vascular disease, diffuse hypoxic/ischemic damage	Moderate	No	Pneumonia, acute respiratory failure, candidosis
#12	80	F	8	Y	4	Cerebral atrophy	Cognitive decline, Alzheimer type	AD neuropathological changes, diffuse hypoxic/ischemic damage	Moderate	No	Pentalobar pneumonia, respiratory insufficiency
#13	81	F	NA	Y	3	NA	NA	Diffuse hypoxic/ischemic damage	Mild	No	Pneumonia, acute respiratory failure
#14	63	F	NA	N	3	No signs	No signs	Diffuse hypoxic/ischemic damage	Mild	No	Ischemic heart disease
#15	70	M	38	Y	5	Chronic ischemic vascular disease	NA	Ischemic vascular disease, diffuse hypoxic/ischemic damage	Moderate	No	Bilateral pneumonia, respiratory insufficiency
#16	81	M	10	Y	4	Cerebral atrophy	Cognitive decline, Alzheimer type	Mixed AD neuropathological changes and Lewy Body pathology, diffuse hypoxic/ischemic damage	Moderate	No	Pneumonia, respiratory insufficiency
#17	75	M	58	Y	6	Cerebral atrophy	NA	Vascular dementia, ischemic vascular disease, diffuse hypoxic/ischemic damage	Moderate	No	Pneumonia, respiratory insufficiency
#18	87	M	30	Y	6	Cerebral atrophy	Cognitive decline	AD neuropathological changes, diffuse hypoxic/ischemic damage	Moderate	No	Pneumonia, multiorgan failure

### A wide spectrum of neuropathological alterations were detected in both COVID-19 and control subjects

The brains of 20 COVID-19 subjects displayed gross macroscopic abnormalities including mild-to-moderate generalized cerebral atrophy (*N* = 9), diffuse cerebral edema (*N* = 9) and chronic territorial ischemic injury (*N* = 6) (Fig. 1b–d). Histopathological evaluation revealed diffuse hypoxic/ischemic damage as a common finding in the COVID-19 cohort, with most subjects presenting mild-to-moderate diffuse hypoxic/ischemic damage of the cerebral hemispheres and brainstem (Fig. 1c), quantified according to a four-tiered semi-quantitative scale (reported in Table 1). Furthermore, acute ischemic injuries were evident in five patients. Small vessels were congested in most subjects, with moderate perivascular extravasation at the level of the medulla, pons and deep cerebellar nuclei in six cases. Variable degrees of astrogliosis were evident in all subjects in all assessed regions, but were more pronounced at the level of the brainstem, as testified by GFAP staining (Supplementary Fig. 2; semi-quantitative evaluation of astrogliosis across brain regions is available in Supplementary Table 2). Alzheimer Disease (AD) neuropathological changes, evaluated according to NIA-AA criteria, as well as Cerebral Amyloid Angiopathy (CAA) were detected in five subjects. In one case, Parkinson’s Disease neuropathological alterations (i.e., pallor of the ventrolateral substantia nigra and nigral lewy bodies) were found.

Control subjects presented similar macroscopic and histopathological alterations: mild-to-moderate generalized cerebral atrophy (*N* = 7), mild-to-moderate diffuse cerebral edema (*N* = 11) and chronic territorial ischemic injury (*N* = 7); most subjects who died due to pneumonia or respiratory failure presented variable degrees of diffuse hypoxic/ischemic damage, with mild to moderate damage of the brainstem being a common finding, similarly to COVID-19 subjects (Fig. 1c); individual findings for hypoxic/ischemic injury are reported in Table 2. Four subjects presented AD neuropathological changes and CAA, with one subject presenting both AD and Lewy Body Dementia mixed pathology. The macroscopic and histopathological findings of both COVID-19 subjects and controls are reported in Tables 1 and 2.

### CNS platelet-enriched microthrombi in small parenchymal vessels were detected in COVID-19 subjects, but not in controls

Small vessel thromboses were detected in nine COVID-19 patients at the level of the pons, deep cerebellar nuclei and cerebral cortex, with one patient presenting small vessel thromboses in multiple sites. No CNS or systemic thromboses were detected in controls. In all COVID-19 cases, CD61 immunoperoxidase staining revealed platelet-rich microthrombi in small parenchymal vessels, with no evidence of arachnoid of meningeal vessels being involved, as seen in Fig. 1d. Other organs were often affected, such as the lungs, liver, intestine, and hypopharynx and even the carotid body^10,23,24^, as summarized in Table 1. In three out of nine cases, microthromboses were identified only within the CNS, while in the remaining six subjects, pulmonary thromboses were also detected. Interestingly, three out of nine subjects with CNS microthrombi were on antithrombotic medication, four were not actively treated prior to death, and in two cases clinical information regarding antithrombotic medication was incomplete. In line with previous findings in literature, CNS microthromboses appear to be peculiar to the COVID-19 cohort, with no control subject presenting either fibrin- or platelet-enriched microthrombi in the CNS or other organs regardless of the cause of death.

### RT-PCR analyses of FFPE tissue sections detected viral RNA in COVID-19 cases with viral protein immunoreactivity

**Fig. 2:**
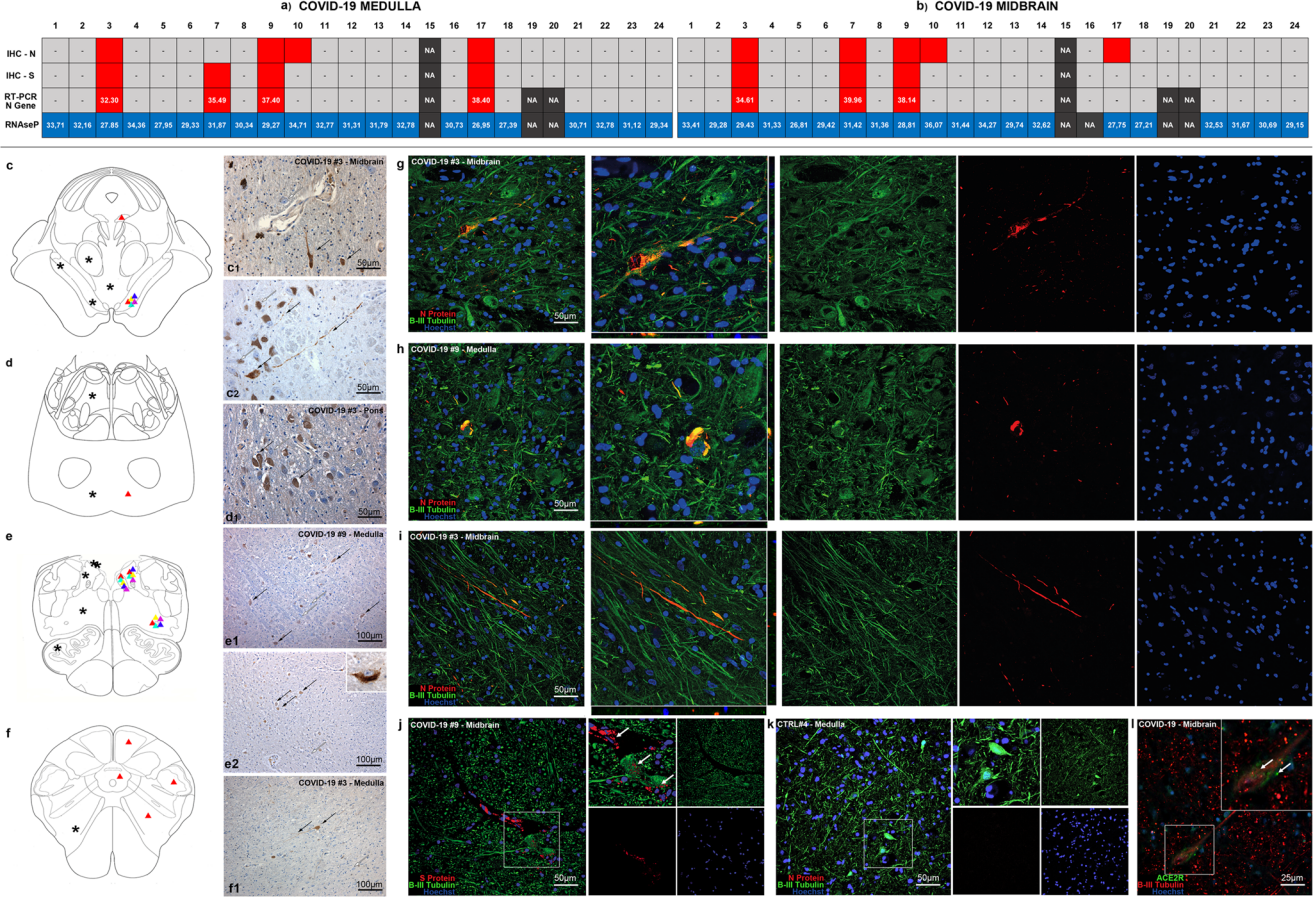
SARS-CoV-2 viral antigens, S and N protein, were detected in the dorsal medulla and in the substantia nigra in five COVID-19 subjects. Topographical localization of SARS-CoV-2 viral protein immunoreactivities (triangles, right half) and microglial nodules (asterisks, left half) throughout the brainstem. **a**, **b** N and S protein IHC, real-time RT-PCR cycle thresholds for SARS-CoV-2 N gene and RNAseP quality control in our COVID-19 cohort at the level of the medulla (L) and midbrain (M). **c** At the level of the midbrain, immunoreactivities are found mainly within the boundaries of the substantia nigra, with the exception of Subject #3, which also presented immunoreactive neurons within the interstitial nucleus of Cajal; microglial nodules were confined mainly within the boundaries of the tegmentum, and were not detected neither within the pes nor the tectum. **c**1 SARS-CoV-2 spike protein IHC at the level of the substantia nigra reveals immunoreactive neurons (mean of two immunoreactivities per mm^2^) with well-marked processes (black arrows); negative neurons can also be found nearby (white arrows). **c**2 SARS-CoV-2 Nucleocapsid Protein IHC reveals a similar pattern of immunoreactive neurons and axons throughout the substantia nigra. **d** At the level of the pons, Subject #3 presented immunoreactive neurons (mean of five immunoreactivities per mm^2^) within the basilary nuclei, while microglial nodules were found both within the basis, as well as the dorsal pons in proximity to the facial nucleus. **d**1 SARS-CoV-2 spike protein IHC at the level of the pons in Subject #3, displaying immunoreactive neurons (black arrows) within the basilary nuclei of the pons; non-reactive cells can also be appreciated (white arrows). **e** At the level of the upper medulla oblongata, immunoreactivities were found at the level of the dorsal motor nucleus of the vagus, solitary tract nucleus and nucleus ambiguus; microglial nodules were prominent within the Vagal Trigone and Area Postrema, but were also found within the reticular formation and the inferior olivary complex. **e**1–2 SARS-CoV-2 spike protein IHC at the level of the solitary tract nucleus and nucleus ambiguus; immunoreactive neurons can be seen within the anatomical boundaries of these nuclei (black arrows), along with non-reactive cells (white arrows). Inset of a single reactive neuron within the solitary tract nucleus, spike protein immunohistochemistry. **f** At the level of the Lower Medulla Oblongata, immunoreactivities were found at the level of the spinal trigeminal nucleus and medullary reticular formation. Microglial nodules were found within the medullary reticular formation. **f**1 SARS-CoV-2 Spike Protein IHC at the level of the medullary reticular formation in the lower medulla (black arrows); non-reactive cells are indicated with a white arrow. Double label N and S protein (red) and Beta-III Tubulin (green) fluorescent immunohistochemistry in COVID-19 subjects (**g**–**j**) and controls (**k**). Distinct immunoreactive neurons and neurites can be appreciated in both the medulla (dorsal motor nucleus of the vagus) (**h**) and midbrain (substantia nigra) (**g**, **i**, **j**). Juxtavascular immunoreactive neurons in proximity to an immunoreactive vessel in the midbrain of COVID-19 subject #9 (**j**, inset). Control subjects present no viral protein immunoreactivity (**k**). **l** Double label ACE2R (green) and Beta-III Tubulin (red) fluorescent immunohistochemistry reveals ACE2R expression of midbrain neurons.

Molecular testing by real-time RT-PCR detected SARS-CoV-2 RNA in 10 out of 24 COVID-19 subjects, 9 of whom had also SARS-CoV-2 S and/or N protein-positive IHC/IF (Fig. 2a, b and Supplementary Table 2). In positive tissue samples, threshold cycles (Ct) of real-time RT-PCR for SARS-CoV-2 RNA ranged between 33 and 38, while in all samples the Ct values of the internal control RNAseP ranged between 27 and 34. The cycle threshold values for each analyzed section are reported in Supplementary Table 2 and in Fig. 2a, b for the medulla and midbrain. SARS-CoV-2 subgenomic RNA was investigated but not detected in our specimens, likely due to RNA degradation within FFPE sections.

### SARS-CoV-2 viral proteins were detected in neurons of the medulla and midbrain in a subset of COVID-19 subjects, but not in controls

**Fig. 3:**
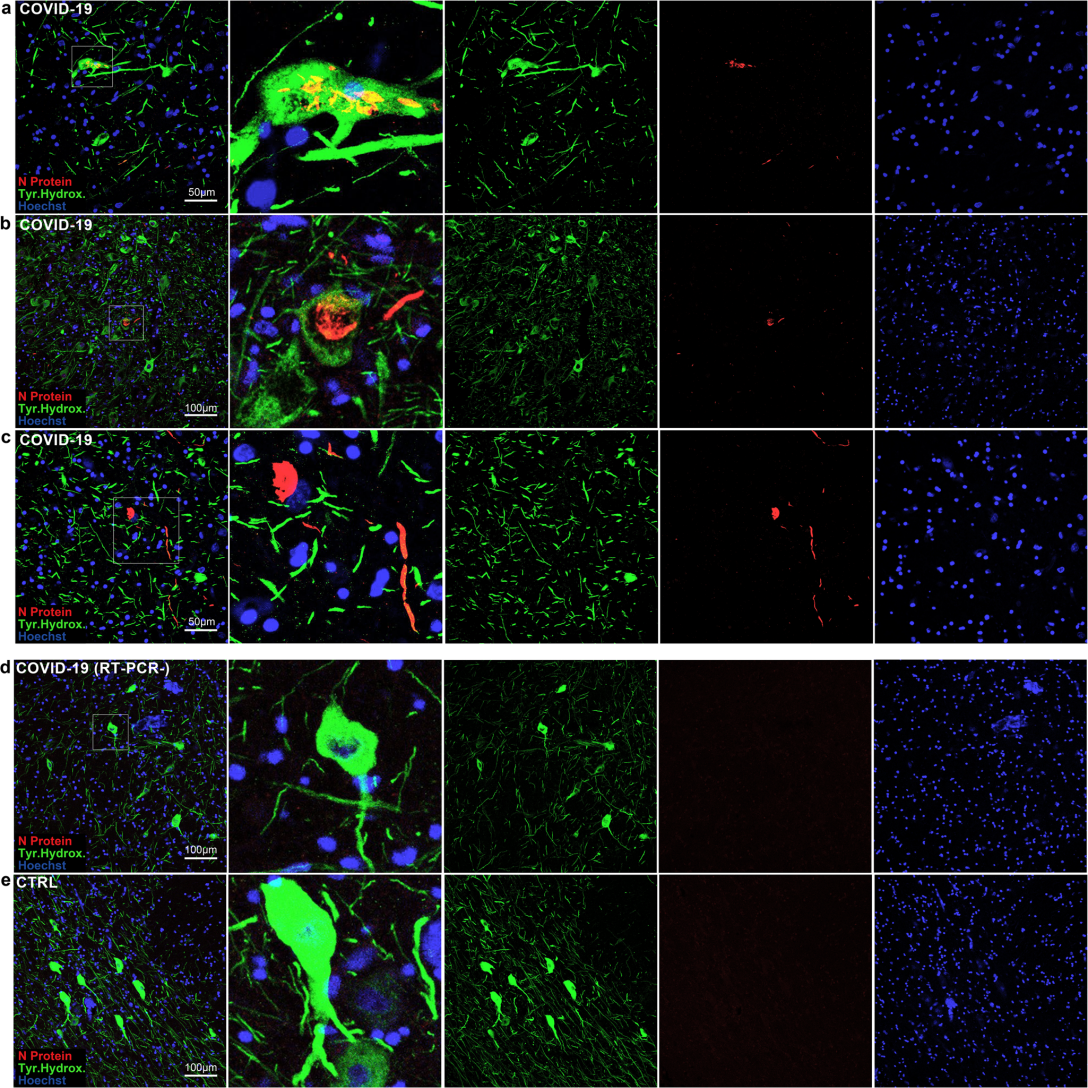
SARS-CoV-2 antigens were detected in both dopaminergic and non-dopaminergic neurons of the substantia nigra. Double label N protein (red) and Tyrosine Hydroxylase (green) fluorescent immunohistochemistry at the level of the substantia nigra in the midbrain. **a**–**c** In COVID-19, Both TH+ and TH− neurons display N protein immunoreactivity. In COVID-19 subjects with negative RT-PCR/IHC (**d**) as well as controls (**e**) no N protein staining was detected.

**Fig. 4:**
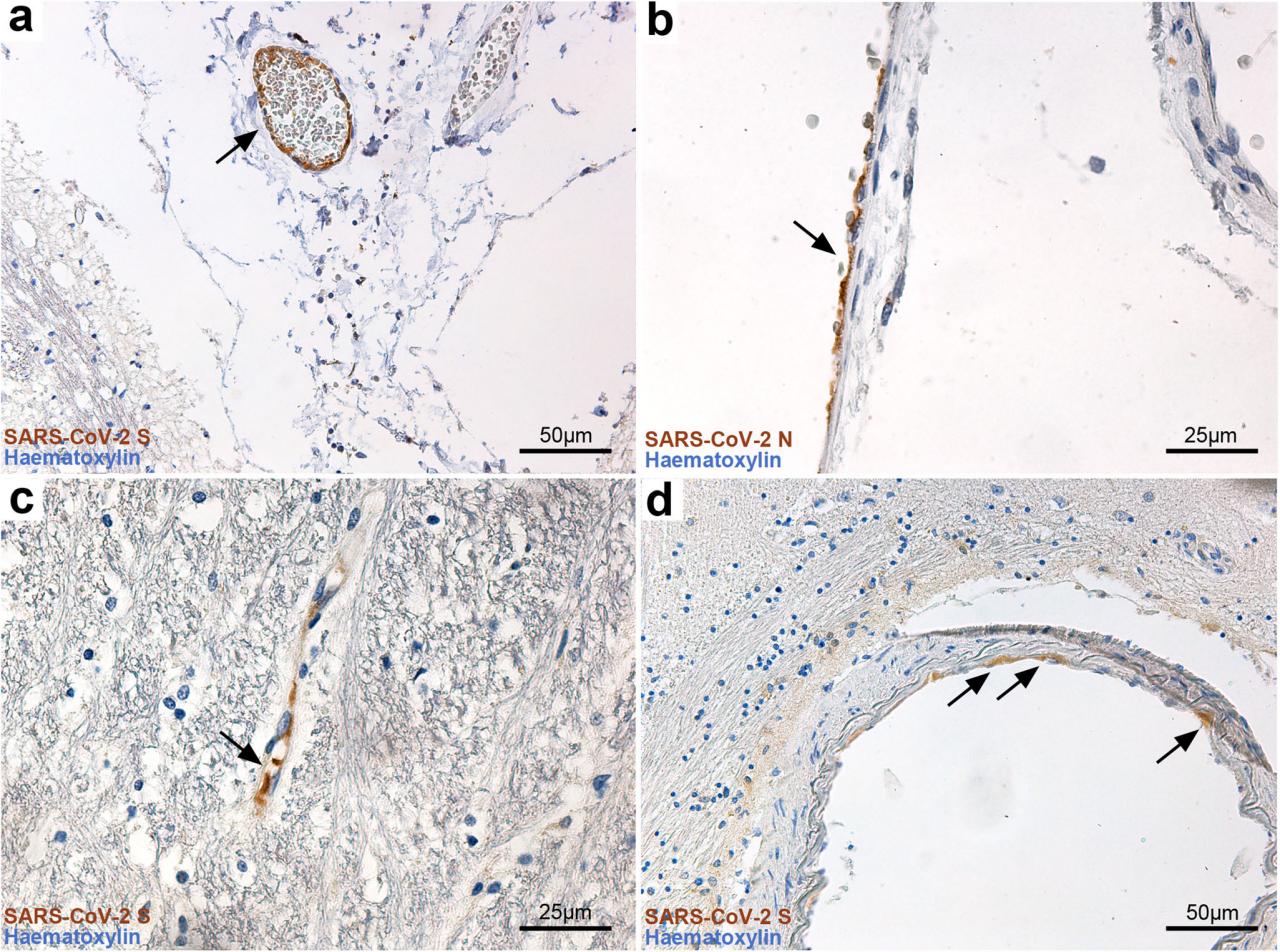
Immunoperoxidase staining for SARS-CoV-2 viral proteins reveals endothelial cell immunoreactivity at the level of the brainstem. **a** SARS-CoV-2 spike protein immunohistochemistry revealing endothelial cell reactivity in a leptomeningeal vessel of the medulla oblongata. **b** SARS-CoV-2 nucleocapsid protein immunohistochemistry in a leptomeningeal vessel in the cerebellum. **c**, **d** SARS-CoV-2 spike protein immunohistochemistry revealing endothelial cell immunoreactivity in the CNS parenchyma.

Immunoperoxidase and immunofluorescent staining for SARS-CoV-2 spike protein and nucleocapsid protein was performed on all samples of included subjects, showing only positive results in cases with SARS-CoV-2 infection, but not in controls, indicating specificity. In particular, viral proteins were detected in seven subjects (#3, #7, #9, #10, #11, #17, #18) within CNS parenchyma and in five subjects (#3, #7, #9, #10, #17) with immunoreactive neurons within the anatomically defined boundaries of the solitary tract nucleus, dorsal motor nucleus of the vagus, nucleus ambiguus and substantia nigra (Fig. 2c–f). As seen in double immunofluorescence labeling, SARS-CoV-2 Nucleocapsid protein antibody can be detected in β-III Tubulin (a pan-neuronal marker) immunoreactive structures, such as neuronal somata and neurites in the medulla and midbrain (Fig. 2g–j), with no labeling in controls (Fig. 2k). At the level of the midbrain, Nucleocapsid protein immunofluorescence was also found within tyrosine hydroxylase immunoreactive neurons and neurites of the substantia nigra, indicating the presence of viral antigens within dopaminergic neurons (Fig. 3a–e and Supplementary Fig. 3). Some of these subjects (#7, #9, #11, #17, #18) also displayed endothelial cell immunoreactivity in small vessels of the cerebral cortex (subject #11), deep cerebellar nuclei (#17–18) hippocampus (#7) (Fig. 4) and midbrain (#9) (Fig. 2j); small vessel thromboses, perivascular extravasation and hemorrhagic injury were found within affected regions of these cases.

In case #7, ischemic injury of the right rostral hippocampal formation due to posterior cerebral artery (PCA) occlusion was associated with perivascular extravasation, edema, fibrinogen leakage and viral protein immunoreactivity within small vessel endothelium, further confirmed by RT-PCR. Acute hemorrhagic injury in the territory of the right middle cerebral artery (MCA) in #11 (Fig. 1b) was associated with endothelitis within perilesional tissue, displaying both viral protein immunoreactive endothelium and positive RT-PCR. Similarly, the deep cerebellar white matter and dentate nuclei in cases #17–18 presented small vessel thromboses and extensive hemorrhagic injury (case #17). Conversely, in some cases with small vessel thromboses within the pons and frontal cortex (e.g., #19–20), viral proteins and RNA was not detectable. ACE2 receptor protein and TMPRSS2 protein immunoreactivity was compatible with the anatomical distribution of SARS-CoV-2 antigens (Fig. 2l and Supplementary Fig. 1E, F). Both proteins were moderately expressed in vascular endothelial cells, brainstem neurons and astrocytes.

### Microglial cells with an activated phenotype and frequent microglial nodules were found in COVID-19 subjects, but not in controls

**Fig. 5:**
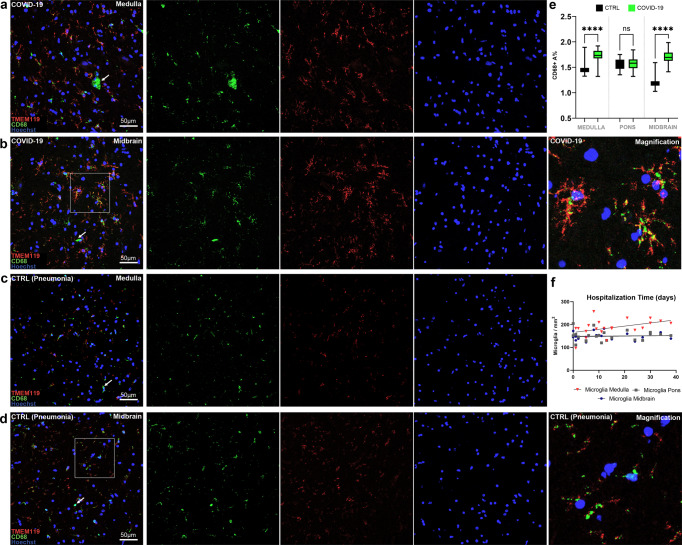
Double label TMEM119 (microglia marker, red)/CD68 (lysosomal activity marker, green) fluorescent immunohistochemistry in COVID-19 and control subjects. **a**, **b** In COVID-19, microglial cells present a distinctly activated phenotype whilst maintaining homeostatic microglial marker TMEM119 (red) and displaying increased lysosomal activity (CD68, green). Arrows indicate CD68+/TMEM119− monocyte/macrophage in the parenchyma. **c**, **d** In control subjects, TMEM119 marks both the soma and sparse ramifications of resident microglia, suggesting less prominent activation without significant marker downregulation. CD68 immunoreactivity (green) is also present, but not as distributed as in COVID-19. **e** Welch one-way ANOVA of CD68+ A% in COVID-19 and controls reveals statistically significant differences between the two groups at the level of the medulla (*p* < 0.0001) and midbrain (*p* < 0.0001), but not the pons. The box plots boundaries represent, from the bottom to the top, the 25th percentile (lower box boundary), the median (central line) and 75th percentile (upper box boundary); whiskers represent the minimum and maximum values. **f** Spearman correlation between microglial densities across brainstem levels and hospitalization time reveals a statistically significant positive correlation between medullary microgliosis and hospitalization time (*r* = 0.44; *p* = 0.044).

In 23 COVID-19 subjects parenchymal microglia displayed an activated phenotype with characteristic thorny ramifications or amoeboid morphology (Fig. 5a, b). Interestingly, homeostatic microglial marker TMEM119 was consistently expressed in our cohort (Fig. 5a–d), even though it is known to be downregulated upon microglial activation in various neuropathological conditions^25^. A similar pattern of immunoreactivity is also seen in Matschke et al.^15^ and Schwabenland et al.^18^. Considering the relatively short hospitalization time prior to death of our COVID-19 cohort (14.5 days), and the similar immunoreactivity pattern compared to other available studies, it could be inferred that TMEM119 downregulation does not occur early in COVID-19.

**Fig. 6:**
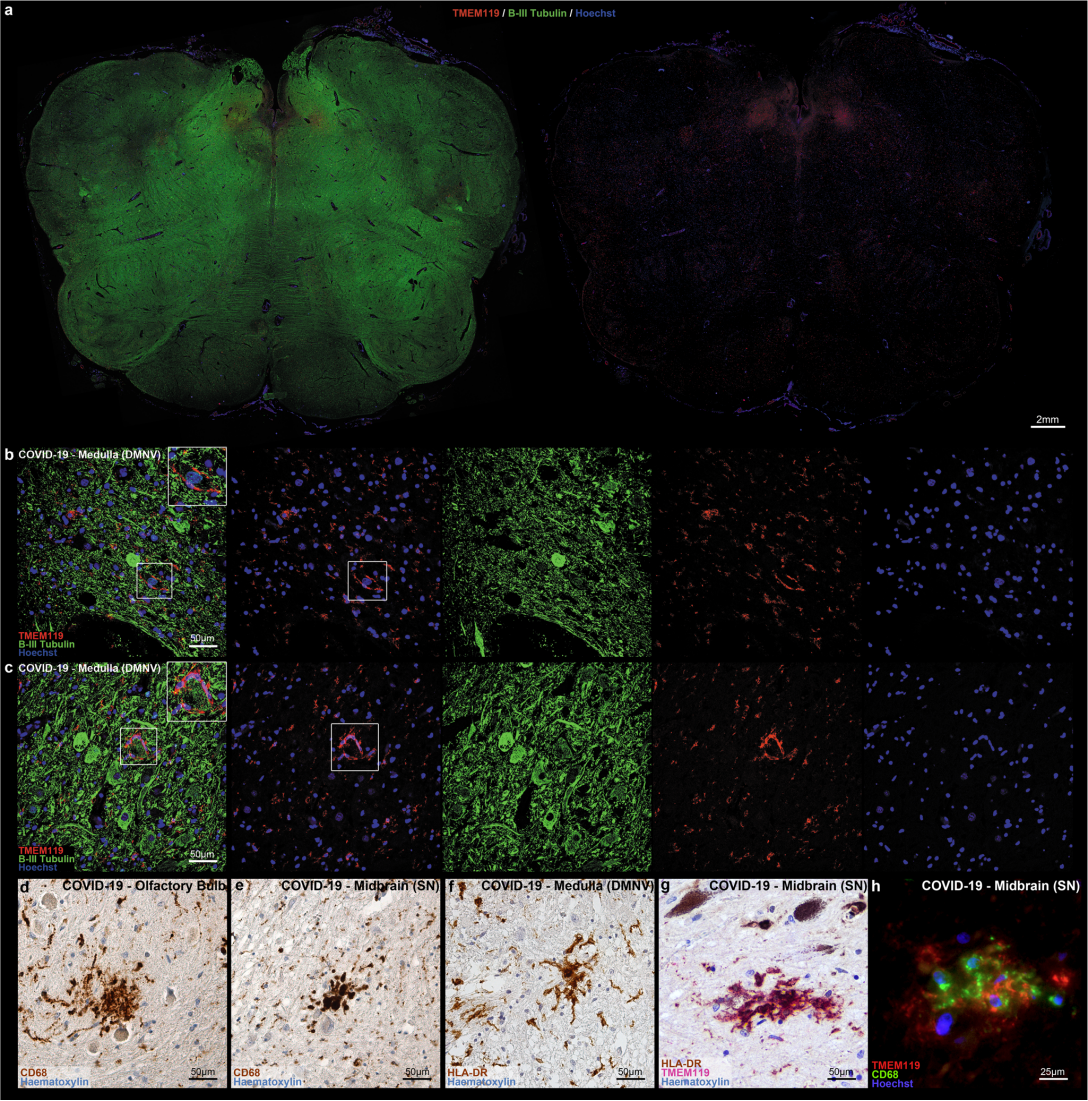
COVID-19 microgliosis and associated microglial nodules. **a** Low-magnification perspective of the medulla oblongata in COVID-19, displaying a ventral-to-dorsal increasing gradient of microglial densities (TMEM119, red; Beta-III Tubulin, green). **b**, **c** Double label TMEM119 (microglial marker, red) and Beta-III Tubulin (neuronal marker, green) immunofluorescent staining at the level of the medulla oblongata. Insets display neuronophagia at the level of the dorsal motor nucleus of the vagus in two COVID-19 subjects. **d**–**h** TMEM119+, CD68+ and HLA-DR+ microglial nodules in the olfactory bulb, medulla oblongata and midbrain of COVID-19 subjects.

While microglial cells expressed homeostatic marker TMEM119 as well as lysosomal-activity marker CD68 in both COVID-19 and control subjects (5A–D), COVID-19 subjects displayed a more widespread CD68+ immunoreactivity (5A–B), with statistically significant differences in CD68 immunoreactive area (expressed as percentage, A%) at the level of the medulla and midbrain, but not the pons, when compared to controls (Fig. 5e, Welch ANOVA *W* = 42.68; medulla *p* < 0.0001; pons *p* = 0.733; midbrain *p* < 0.0001). We also found a positive correlation between medullary microgliosis and hospitalization time, as described in detail in the following paragraphs (Fig. 5f). Ki-67 immunoperoxidase staining, as well as Ki-67/CD68 double label immunofluorescent staining did not reveal significant Ki-67 immunoreactivity ascribable to microglial cells, suggesting local microglial activation and migration without active proliferation in the considered cases. Microglial nodules associated with perineuronal HLA-DR+/TMEM119+/CD68+ cells were suggestive of neuronophagia in 18 COVID-19 subjects (Fig. 6a–h) and were identified at the level of the substantia nigra (*N* = 14), dorsal motor nucleus of the vagus (*N* = 12), medullary reticular formation (*N* = 9), area postrema (*N* = 6) and basal ganglia (*N* = 5); no microglial nodules were found in control cases, regardless of cause of death. Moreover, moderate to severe infiltration of CD68+/TMEM119− cells was found in 23 subjects; given their prominent perivascular localization, these were likely monocyte-derived macrophages.

### COVID-19 subjects and pneumonia controls present CD206 expressing perivascular macrophages with anti-inflammatory M2 phenotype

**Fig. 7:**
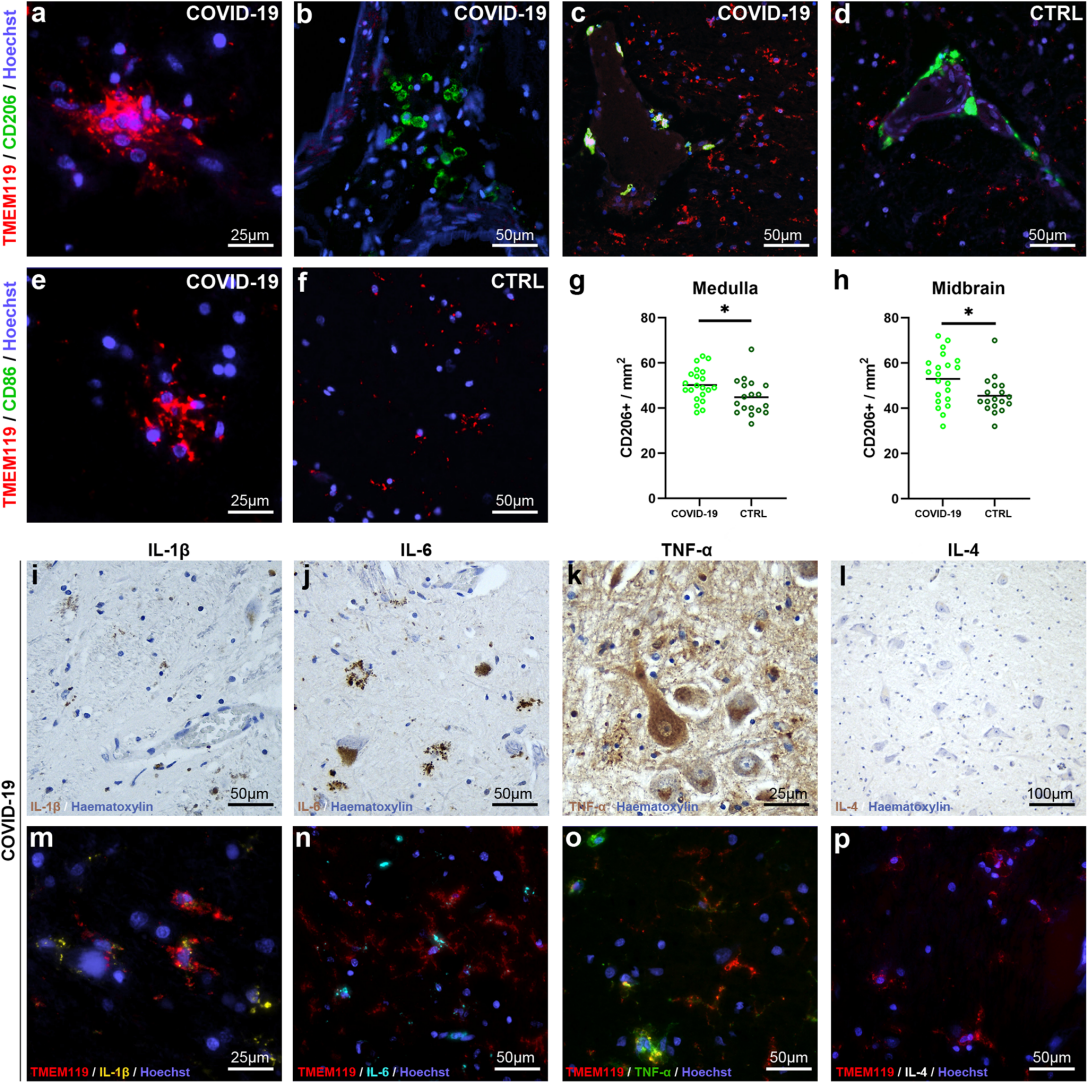
Perivascular macrophages express anti-inflammatory marker CD206, while microglia display immunoreactivity for pro-inflammatory cytokines IL-1β, IL-4, TNF-α but not IL-4. **a** Microglia and microglial nodules do not display CD206 immunoreactivity. **b**–**d** Perivascular monocytes/macrophage-like cells display CD206 immunoreactivity in both COVID-19 and control subjects. **e**, **f** Microglia and perivascular cells do not display immunoreactivity for CD86, in either COVID-19 or control subjects. **g**, **h** Welch corrected *t*-test reveals statistically significant differences between COVID-19 and control CD206+ cell densities in both medulla and midbrain. **i**–**p** Microglial cells are immunoreactive to pro-inflammatory cytokines IL-1β, IL-4, TNF-α but not IL-4.

Evaluation of M1/M2 phenotype markers CD86 and CD206 revealed CD206+ perivascular macrophages in both COVID-19 and control subjects (Fig. 7a, b). Morphometrical quantification of CD206+ perivascular cells revealed statistically significant differences between groups, with COVID-19 subjects presenting higher densities of pro-inflammatory macrophages in both medulla and midbrain (Fig. 7c, d).

### Microglia did not express M1/M2 cell surface markers CD86/CD206, but presented a pro-inflammatory phenotype based on interleukin expression

While the expression of M1/M2 cell surface markers was not detected, TMEM119+ microglial cells displayed immunoreactivity for Interleukin-1β, Interleukin-6, TNF-α but not Interleukin-4, indicating a pro-inflammatory phenotype (Fig. 7k–r). The expression of pro-inflammatory cytokines, but not M1 surface marker CD86, is likely associated with the acute microglial response occurring in the early phases of infection, also testified by the lack of TMEM119 downregulation.

### In COVID-19 subjects, a topographically defined pattern of microgliosis was found in the medulla oblongata and midbrain

**Fig. 8:**
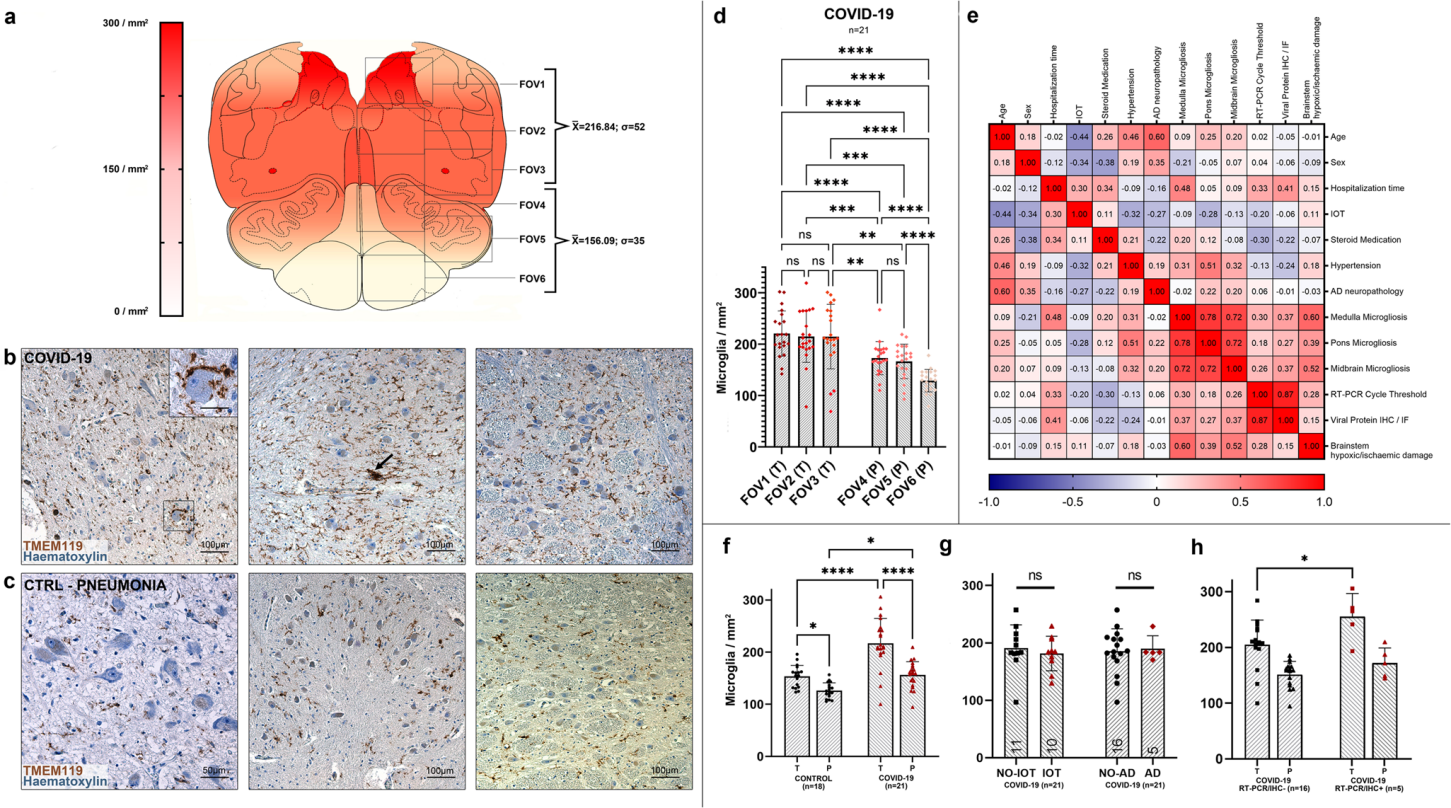
Quantification of reactive microglia at the level of the medulla oblongata reveals an anatomically segregated pattern of inflammation. **a** Anatomical heatmap of activated microglia within the medulla oblongata in COVID-19. **b**, **c** TMEM119 immunoperoxidase staining of comparable regions of the medulla oblongata in COVID-19 subjects (above) and controls (below). Inset: neuronophagia in the dorsal motor nucleus of the vagus. Arrow: microglial nodule. **d** Welch one-way ANOVA of microglial densities per counting fields (FOV) reveals statistically significant differences between FOVs of the Tegmentum (T; FOV1–3) when compared to FOVs of the Pes (P; FOV4–6). **e** Correlation heatmap between COVID-19 subject clinical data and neuropathological findings. **f** Welch one-way ANOVA between anatomical compartments (T, tegmentum; P, pes) between COVID-19 subjects (*n* = 21, red) and controls (*n* = 18, black) reveals statistically significant differences both at the level of the medullary tegmentum (*p* < 0.0001) and pes (*p* = 0.017). **g** Welch corrected *t*-test plot of microglial densities (microglia/mm^2^) in the medulla oblongata of COVID-19 subjects treated (*n* = 10, red) and not treated (*n* = 11, black) with intensive oxygen therapy (*p* > 0.05), and of COVID-19 subjects with (*n* = 5, red) and without (*n* = 16, black) Alzheimer’s disease neuropathological changes (*p* > 0.05). **h** Welch one-way ANOVA between anatomical compartments (T, tegmentum; P, pes) between COVID-19 subjects with (*n* = 5, red) and without (*n* = 16) viral tropism (RT-PCR/IHC+ versus RT-PCR/IHC−) reveals statistically significant differences at the level of the medullary tegmentum (*p* = 0.017). Error bars indicate standard deviation.

At the level of the medulla oblongata, a topographically defined pattern of microgliosis was detected, as seen in Fig. 8a–c. Welch one-way ANOVA of individual counting fields (FOVs) (Fig. 8d) revealed statistically significant differences (*p* < 0.001) in TMEM119+/CD68+ activated microglial cell densities between the medullary tegmentum (T, FOV-13; 216.84 ± 52.26 microglia/mm^2^) and the ventral medulla (pes, P, FOV4–6; 156.09 ± 35.16 microglia/mm^2^). No differences were found between individual counting fields of the same anatomical compartment.

When comparing microglial densities between COVID-19 patients and the control cohort, statistically significant differences were found when considering overall medullary microgliosis, as well as single anatomical compartments (Fig. 8f). Furthermore, no significant differences were found when comparing microglial densities between IOT and non-IOT COVID-19 patients, as well as AD and non-AD patients with SARS-CoV-2 infection (Fig. 8g).

**Fig. 9:**
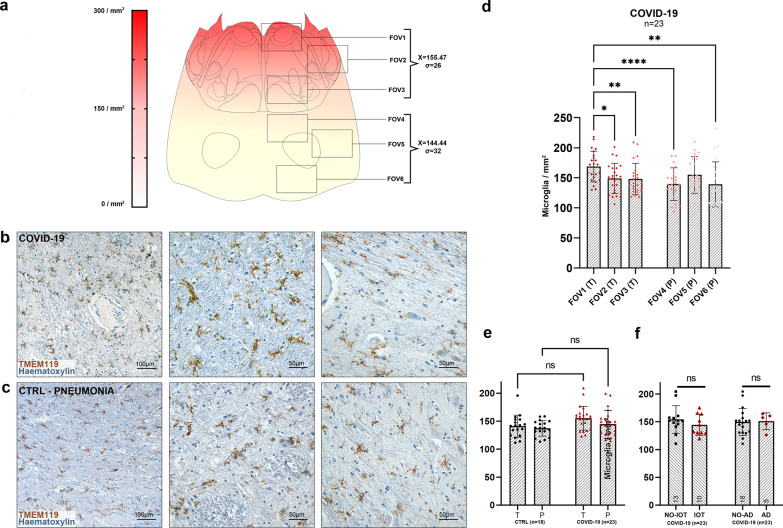
No statistically-significant differences in microglial densities were found at the level of the pons. **a** Anatomical heatmap of activated microglia within the pons in COVID-19. **b**, **c** TMEM119 immunoperoxidase staining of comparable regions of the pons in COVID-19 subjects (above) and controls (below). **d** Welch one-way ANOVA of microglial densities per counting fields (FOV) reveals statistically significant differences between FOV1 (dorsal pons, including locus coeruleus) with other pontine counting fields. **e** Welch one-way ANOVA between anatomical compartments (T, tegmentum; P, pes) between COVID-19 subjects (*n* = 23, red) and controls (*n* = 18, black) reveals no statistically significant differences either at the level of the medullary tegmentum (*p* = 0.55) and pes (*p* = 0.98). **f** Welch corrected *t*-test plot of microglial densities (microglia/mm^2^) in the pons of COVID-19 subjects with (*n* = 5, red) and without (*n* = 16, black) Alzheimer’s disease neuropathological changes (*p* > 0.05), and of COVID-19 subjects treated (*n* = 10, red) and not treated (*n* = 13, black) with intensive oxygen therapy (*p* > 0.05). Error bars indicate standard deviation.

At the level of the pons, a less pronounced pattern of regional microgliosis was identified, as shown in Fig. 9a–c. Welch one-way ANOVA of individual counting fields revealed statistically significant differences only between the most dorsally located counting field comprising the locus coeruleus (FOV1), and other counting fields (FOV2–6) (Fig. 9d). Differences in overall microgliosis, as well as differences between anatomical compartments, were not significant between COVID-19 subjects and controls (Fig. 9e). As for the medulla, no differences were found between IOT and non-IOT patients, as well as for AD versus non-AD patients (Fig. 9f). Hence, while there appears to be a higher degree of microgliosis in proximity to the locus coeruleus in COVID-19 when compared to other regions of the pons, no differences were found within COVID-19 subgroups and when compared to controls.

**Fig. 10:**
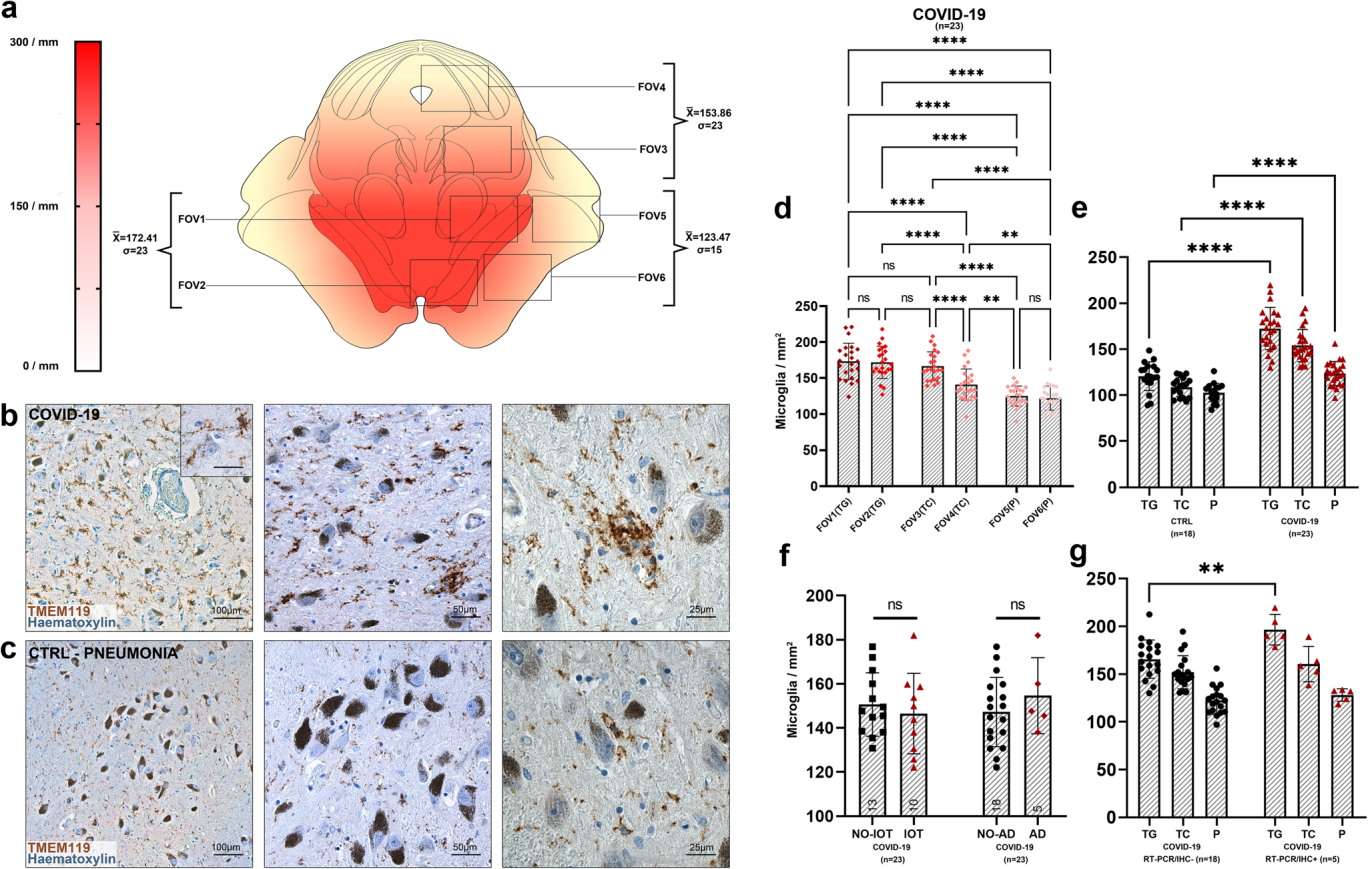
Anatomically segregated microgliosis of the ventral midbrain was significantly higher in COVID-19 when compared to pneumonia patients. **a** Anatomical heatmap of activated microglia within the medulla oblongata in COVID-19. **b**, **c** TMEM119 immunoperoxidase staining of comparable regions of the midbrain in COVID-19 subjects (above) and controls (below). Inset: perineuronal microglia in the substantia nigra. COVID-19 subjects often present distinct microglial nodules. **d** Welch one-way ANOVA of microglial densities per counting fields (FOV) reveals statistically significant differences between FOVs of the Tegmentum (T; FOV1–2) when compared to FOVs of the Pes (P; FOV5–6), suggesting for a localized pattern of microgliosis comprising the preacqueductal tegmentum and the substantia nigra. **e** Welch one-way ANOVA between anatomical compartments (TG, tegmentum; TC, tectum; P, pes) between COVID-19 subjects (*n* = 23, red) and controls (*n* = 18, black) reveals statistically significant differences between all anatomical districts of the midbrain (*p* < 0.0001). **f** Welch corrected *t*-test plot of microglial densities (microglia/mm^2^) in the midbrain of COVID-19 subjects with (*n* = 5, red) and without (*n* = 16, black) Alzheimer’s disease neuropathological changes (*p* > 0.05), and of COVID-19 subjects treated (*n* = 10, red) and not treated (*n* = 11, black) with intensive oxygen therapy (*p* > 0.05). **g** Welch one-way ANOVA between anatomical compartments (TG tegmentum, TC tectum, P pes) between COVID-19 subjects with (*n* = 5, red) and without (*n* = 18) viral tropism (RT-PCR/IHC+ versus RT-PCR/IHC−) reveals statistically significant differences at the level of the midbrain tegmentum (*p* = 0.0074), but not other anatomical districts. Error bars indicate standard deviation.

At the level of the midbrain, COVID-19 subjects presented marked topographical differences in microglial densities between counting fields comprising the substantia nigra (midbrain tegmentum, FOV1–2 and FOV3) when compared to counting fields of the midbrain tectum and pes (FOV4–6), as seen in the anatomical heatmap in Fig. 10a–c and by Welch one-way ANOVA in Fig. 7d. This anatomically segregated pattern of inflammation targeting mainly the substantia nigra, but also part of the pre-acqueductal tegmentum, indicates an increasing dorsal-to-ventral gradient of microgliosis which affects the gray matter of the midbrain, sparing counting fields falling within the cerebral peduncle (FOV5–6). When compared to controls, COVID-19 subjects presented significantly higher microglial densities when considering both overall microgliosis, as well as microglial densities within anatomical compartments (Fig. 10e), suggesting for a COVID-19-specific microglial response at the level of the midbrain. Similar to other brainstem levels, no statistically significant differences in overall microgliosis were found when comparing IOT and non-IOT subjects, as well as AD and non-AD subjects (Fig. 10f).

We also found a strong correlation between microglial densities across the different levels of the brainstem, as well as CD68+ A% of the corresponding level. The strong positive correlation between microglial density and CD68 immunoreactive area further underlines the activated phenotype displayed by microglial cells in COVID-19. Interestingly, in the COVID-19 cohort, hospitalization time was positively correlated to microglial density in the medulla (*r* = 0.44; *p* = 0.044), but not with microglial density in the pons and midbrain (Fig. 5f). This appears to indicate an increase of microglial densities in the medulla as infection progresses, while the levels of microgliosis within the rest of the brainstem appear to remain relatively stable throughout time. Considering the numerous instances of microglial nodules and neuronophagia encountered in the medulla of the COVID-19 cohort, this is suggestive of prominent medullary impairment ongoing during COVID-19, regardless of oxygenation status or prior neurodegenerative pathology. Conversely, microglial density in the medulla also correlates with hypoxic/ischemic damage of the brainstem, evaluated along a four-tiered semi-quantitative scale (*r* = 0.59; *p* = 0.004), as also seen in Thakur et al.^19^. Hence, while microgliosis is strongly characteristic of COVID-19 subjects and differs from controls, brainstem hypoxia/ischemia plays a major role in mediating medullary microgliosis, as seen in our cohort and in accordance to previous literature.

### Viral antigens are associated to higher microglial densities within affected anatomical loci, but no differences are found in overall microgliosis, suggesting a specific topographical response

While overall levels of microgliosis within the medulla, pons and midbrain did not differ significantly between COVID-19 subjects with and without detectable viral antigens, Welch one-way ANOVA between anatomical compartments (i.e., tegmentum, tectum and pes) revealed statistically significant differences within the COVID-19 cohort. Indeed, subjects with detectable viral genomic sequences and antigens (RT-PCR+/IHC+) were characterized by higher microglial densities in the medullary (*p* = 0.017) and midbrain tegmentum (*p* = 0.0074) when compared to negative (RT-PCR−/IHC−) COVID-19 subjects, as seen in Figs. 8h and 10g. In association with frequent instances of microglial nodules and perineuronal TMEM119+/CD68+ microglial cells suggestive of neuronophagia (Fig. 6a–h), this finding suggests a peculiar microglial response toward anatomical loci of the brainstem in which SARS-CoV-2 antigens were detected, even though overall levels of microgliosis within brainstem regions did not appear to differ significantly. Taken together with little-to-no Ki-67 immunoreactivity and no detectable Ki-67+/CD68+ immunofluorescent signal, migration of microglial cells toward the site of injury appears to be the more likely mechanism occurring in COVID-19 inflammation, rather than microglial proliferation within affected regions.

## Discussion

In the present study, the neuropathological findings of 24 COVID-19 patients were examined and compared with age- and sex-matched controls who died due to pneumonia and/or respiratory insufficiency. Our findings indicate specific neuropathological alterations in the brains of COVID-19 patients, with particular regard to topographically-defined microgliosis in the brainstem and viral immunoreactivity in specific CNS compartments, either within the boundaries of brainstem nuclei or in the context of ischemic and hemorrhagic injuries. Platelet and fibrin microthrombi, in particular, were characteristic findings of the COVID-19 cohort, and often affected multiple organs, such as the lungs, liver, intestine, hypopharynx and even the carotid body^26–28^, as summarized in Table 1. Microthromboses were more frequent within the pons, deep cerebellar nuclei and cerebral cortex. In some cases, hemorrhagic injury and microthromboses were found in regions with viral protein immunoreactivity in vascular endothelial cells.

SARS-CoV-2 viral proteins, on the other hand, were confined to specific loci of the CNS. As seen in Fig. 2c–j, SARS-CoV-2 viral proteins appear to be localized preferentially within neurons of the vagal nuclei of the medulla and the substantia nigra, with the exception of one subject who also presented immunoreactive cells throughout the whole brainstem (#3). While Matschke et al.^15^ reported SARS-CoV-2 invasion of cranial nerves IX-X, we were unable to replicate these findings within our cohort; furthermore, unlike Meinhardt et al.’s findings^16^, viral proteins and RNA were not detectable in any of the sampled olfactory bulbs, tracts and bifurcations, even though moderate edema, moderate-to-severe astrogliosis and moderate microglial activation were encountered in most cases in our study. ACE2 Receptor and TMPRSS-2 protein immunohistochemistry support this topographical localization, with neurons within the dorsal motor nucleus of the vagus, solitary tract nucleus, nucleus Ambiguus and Substantia Nigra being moderately immunoreactive (Fig. 2l and Supplementary Fig. 1E, F).

While previous studies identified viral protein immunoreactivity in sparse cells throughout the brainstem^15,18^ without specific topography, our findings appear to be in line with available animal studies on other coronaviruses, i.e., SARS-CoV and MERS-CoV, which are known to be able to infect the brainstem, and particularly the dorsal motor nucleus of the vagus, solitary tract nucleus and nucleus ambiguus, so that an analog pattern of neuroinvasion for SARS-CoV-2 has been suggested^14,23,24,29^. The peculiar and unexpected finding in our cohort was the detection of viral proteins and genomic sequences in the substantia nigra, not matching any known models of coronavirus neurotropism. Interestingly, SARS-CoV-2 S and N protein were detected in both tyrosine hydroxylase positive and negative neurons (Fig. 3). SARS-CoV-2 immunoreactive neurons of the substantia nigra were frequently found in proximity to blood vessels, which in the reported instances were immunoreactive to viral proteins as well (Figs. 2j and 4). Hence, aside from olfactory-transmucosal transmission^16^, and vagus/glossopharyngeal-mediated invasion^15^, SARS-CoV-2 may gain access to other districts of the CNS either through a yet-unknown neuronal route or, as suggested by our findings in the midbrain, by crossing the blood-brain-barrier and infecting structures of the peri- and juxtavascular compartment^18^. In literature, SARS-CoV-2 infection of dopaminergic neurons was investigated in vitro, but was not reported in human post-mortem studies to date. Yang et al.^30^ demonstrated that a Spike-enabled pseudo-entry virus is able to infect dopaminergic neurons in an experimental platform of cell and organoid derivatives from human pluripotent stem cells (hPSCs), suggesting SARS-CoV-2 affinity for dopaminergic neurons. Jacob et al.^9^ also detected SARS-CoV-2 tropism in human-induced pluripotent stem cell-derived midbrain organoids, however viral infection and replication was predominant in the choroidal plexus; on the other hand, previous studies of our group failed to demonstrate SARS-CoV-2 tropism in human brain organoids^8^. Hence, even though in vitro studies have produced contradictory results concerning SARS-CoV-2 tropism for the CNS and dopaminergic neurons, neuropathological findings support this hypothesis^17^. Together, the detection of viral proteins in the substantia nigra and the vagal nuclei support the notion that viral infections, such as SARS-CoV-2, may predispose, or quickly precipitate, the development of neurodegenerative diseases, especially Parkinson’s Disease^31–33^. Intriguingly, recent evidence points toward an antiviral role of alpha-Synuclein (αSyn), the protein whose pathological conformations are involved in PD and Multiple System Atrophy (MSA), in response to infection of peripheral tissues. Mice lacking αSyn expression exhibit markedly increased viral growth in the brain, increased mortality and increased neuronal cell death, suggesting a role for αSyn in the neuronal innate immune response. Beatman et al.^34^ documented that native neuronal expression of αSyn inhibited viral infection in the central nervous system, while virus-induced increased αSyn expression localized to endoplasmic reticulum-derived membranes, modulated virus-induced endoplasmic reticulum stress signaling, and inhibited viral replication, growth, and injury in the CNS. Similarly, another study evidenced that αSyn is required for neuronal expression of interferon-stimulated genes and supports neuron-specific interferon responses^35^. Together, these findings suggest a prominent role of αSyn protein, as well as virus-induced increased expression of αSyn, in mediating antiviral responses to CNS infections. Conversely, while αSyn in the brain may represent a protective factor in response to viral infections, its viral-induced overexpression can potentially lead to protein misfolding, aggregation and development of Lewy-body pathology. Indeed, Wu et al.^36^ have demonstrated that SARS-CoV-2 proteins S and N present high binding affinity to αSyn. Furthermore, the authors demonstrated that αSyn upregulation and aggregation was accelerated by SARS-CoV-2 viral proteins, and that overexpression of αSyn led to the development of Lewy-body pathology in vitro. Hence, while αSyn expression increases in neurons in response to infection, the presence of viral proteins can potentially trigger protein misfolding and aggregation as seen in in vitro studies, especially in conjunction with inflammatory, environmental and genetic facilitators, leading toward, or predisposing to, synucleinopathies and neurodegeneration^37,38^.

This hypothesis arises, and is clinically driven, both from the 1917 Spanish flu and von Economo’s encephalitis lethargica pandemics, which have seen a surge of post-encephalitic parkinsonism following the waves of the pandemic, and the known association between viral infection and the development of transient or permanent movement disorders^39^. In fact, pathogens, and in particular respiratory viruses, have been suggested as a potential etiopathogenic factors for PD, leading to parkinsonism in subjects over the age of 50, regardless of genetic substrate^31,40^. Both the olfactory bulb and tract, as well as the medulla oblongata where the vagal nuclei are located, represent the very first sites of early PD neuropathology, and interestingly also represent the main sites of inflammation / infection encountered in COVID-19^41,42^, as seen in our study. Furthermore, viral-related inflammation might render the CNS susceptible to preceding or subsequent stressors^43^, even in the absence of direct viral invasion; indeed, past history of infection was associated with a 20% higher risk of presenting PD in the future^44^.

We believe these findings encourage further research on the possibility that COVID-19 neuroinflammation may be the trigger of a neurodegenerative process, such as Parkinson disease, in susceptible individuals. Future studies on COVID-19 survivors and Long COVID patients are therefore warranted^43^.

However, despite the detection of viral proteins and genomic sequences in restricted regions of the brainstem, we found no evident neuropathological alterations in SARS-CoV-2 infected cells, such as necrotic changes and other cytological alterations, that could hint toward possible direct consequences of viral invasion in human neurons. COVID‐19 is characterized by different evolutionary phases and heterogeneous individual responses, and the short interval between infection and death in our cohort (mean hospitalization time = 14 days), as well as the fact that included patients died during the acute phase of the disease, may not be sufficient to determine detectable neuropathological alterations in affected cells as a direct consequence of viral invasion, which may require more time to develop^3,29^. Lastly, the detection of viral proteins in a subset of patients (5 out of 24), as seen in this and previous studies may be related to the particularly severe disease, and concurring comorbidities, of the patients who underwent neuropathological examination.

Hence, while the consequences of SARS-CoV-2 neurotropism in the medulla have been widely discussed in literature and are supported by the detection of viral proteins and genomic sequences in our study, the absence of direct neuronal damage and the impossibility of performing functional assays on post-mortem samples should be taken into consideration when discussing the clinical implications of COVID-19 neuropathology. Future studies on “long COVID” patients^3^ may be able to shed a light on the long-term consequences of COVID-19, particularly concerning the detection of SARS-CoV-2 within the CNS after the acute phase of the disease, and whether or not this leads to specific neuropathological alterations as a consequence of viral invasion.

Concerning microglial activation and density, our findings appear to be in line with Schwabenland et al.^18^, who identified microglial nodules and parenchymal reactive microglia as hallmark for COVID-19, in contrast to both controls and Extra Corporeal Membrane Oxygenation (ECMO) patients. In our cohort, patients with pneumonia and/or respiratory failure served as control group and, although also characterized by microglial activation, these patients displayed lower microglial counts in the medulla and midbrain, but not in the pons, when compared to COVID-19 subjects. We also found no significant effect of oxygen therapy on microglial density within the COVID-19 group. Conversely, Deigendesh et al.^45^ found significant differences in HLA-DR+ activated microglia when comparing COVID-19 subjects to non-septic controls, but no differences were found with patients who had died under septic conditions; according to the authors^45^ this may represent a histopathological correlate of critical illness-related encephalopathy, rather than a COVID-19-specific finding. Aside from the distinct populations serving as control subjects, significant methodological differences between these studies must be taken into consideration. Our approach to microglial quantification was more similar to Schwabenland et al.^18^, as digitally-assisted manual counting of TMEM119+ cells, a homeostatic microglia-specific marker, was performed to estimate microgliosis. Conversely Deigendesh et al.^45^ quantified HLA-DR immunoreactive area, a marker expressed on both microglia and on infiltrating lympho-monocytic cells, as a fraction of the counting field (A%), and not as individual particles, explaining differences between our studies.

Interestingly, perivascular macrophages were found to express CD206, indicating an anti-inflammatory M2 phenotype. Higher CD206+ cell densities were detected in the perivascular compartment of COVID-19 brains compared to pneumonia controls, in both the medulla and midbrain. Conversely, while microglial cells did not express either CD86 nor CD206, surface markers for M1/M2 phenotypes, expression of IL-1β, IL-6, TNF-α but not IL-4 suggests for a pro-inflammatory phenotype that may precede expression of specific surface markers. This is in line with the lack of TMEM119 downregulation, as seen in the early and acute stages of inflammation.

Interestingly, while no evidence of direct neuronal damage was found in SARS-CoV-2 infected cells, microglial densities within affected anatomical loci differed between subjects with and without detectable viral antigens and genomic sequences (RT-PCR/IHC+ versus RT-PCR/IHC− in Figs. 8h and 10g), suggesting a link between the detection of SARS-CoV-2 antigens and microglial response. Conversely, overall microglial density (i.e., without topographical delineation) did not differ between the two groups, and a strong correlation between microgliosis and hypoxic/ischemic damage at the level of the brainstem was found. Hence, while we found a suggestive link between microgliosis and the detection of SARS-CoV-2 antigens in our cohort, other factors such as hypoxia/ischemia and systemic inflammation/cytokine storm ongoing during COVID-19, as previously reported by Thakur et al.^19^, are likely to play a more prominent role in determining brainstem microgliosis, in accordance to previous studies^45^.

In conclusion, the present study contributes to define the spectrum of neuropathological alterations in COVID-19, as well as the neuroinvasive potential of SARS-CoV-2 within the CNS, with particular regard to its implications for neurodegenerative diseases. Unlike previous findings, we have documented a subset of COVID-19 cases in which viral proteins and genomic sequences were detectable within anatomically defined regions of the CNS. Similarly, microglial activation in the brainstem appears to differ between COVID-19 and pneumonia/respiratory failure controls, with the former also presenting a pattern of increased microglial density in specific compartments of the medulla and midbrain. However, despite this evidence supporting the neuroinvasive potential of SARS-CoV-2, neuropathological alterations encountered in our cohort cannot be ascribed to viral antigens detected in the brainstem. In line with other studies in literature, hypoxic/ischemic damage and systemic inflammation likely represent major contributors in determining neuropathological alterations in COVID-19, with little-to-no evidence indicating direct viral damage of the central nervous system in humans. Moreover, further investigation is required to determine whether or not SARS-CoV-2 neurotropism represents a major component of COVID-19 in the general population, as subjects included in neuropathological studies often present a much more severe course of the disease and major medical comorbidities. Nevertheless, the findings of our study suggest the possibility that, although not frequently, SARS-CoV-2 may gain access to specific regions of the central nervous system, especially the vagal nuclei of the medulla and the substantia nigra in the midbrain. As direct neuropathological alterations determined by SARS-CoV-2 neurotropism may not be detectable in subjects deceased during the acute phase of the disease, future studies are required to determine whether or not SARS-CoV-2 neurotropism is present in chronic COVID-19 patients, or in COVID-19 survivors suffering from the long-term effects of infection, and if eventual neuropathological alterations in these subjects can be ascribed to viral tropism, rather than immune-mediated mechanisms. The possibility that COVID-19 neuroinflammation may trigger or exacerbate pre-existing neurodegenerative conditions especially Parkinson’s disease must therefore be taken into serious consideration.

### Limitations of the study

This study is based on post-mortem tissue samples obtained during the first wave of the COVID-19 pandemic in Italy. While the neuropathological alterations encountered in our work contribute to define the pathological mechanisms of COVID-19 and SARS-CoV-2 infection in the CNS, the lack of exhaustive post-infection neurological evaluation of included patients does not allow for unequivocal clinico-pathological correlations. It must also be considered that most patients included in the study died during the peak of the sanitary emergency in Italy, one of the first countries to face the COVID-19 pandemic in Europe, and neurological evaluation was not always possible. Hence, it remains to be determined whether the neuropathological alterations observed in this study are also linked to neurological symptoms, and whether they are also present in COVID-19 survivors.

Unlike previous studies in literature, we have included 18 controls who died due to pneumonia, respiratory insufficiency or multiorgan failure, rather than healthy controls. Retrospective selection of control subjects, however, could lead to unwanted selection bias. Furthermore, from the available clinical data of our controls, we have found no instances of intensive oxygen therapy or mechanical ventilation, but incompleteness of available clinical records cannot entirely be excluded. For this purpose, we have also performed comparisons within the COVID-19 group, identifying no statistically significant differences between subjects with and without neurodegenerative conditions, and no influence of oxygen therapy on brainstem microgliosis. The involvement of other brain regions, such the cerebral and cerebellar cortex and the basal ganglia, cannot be excluded but is beyond the scopes of this study. Moreover, as all patients died during the first wave of the COVID-19 pandemic, our findings may not reflect the possible neuropathological alterations encountered in patients affected by SARS-CoV-2 variants.

Limitations to viral antigen/RNA detection in our study must also be considered. Real-time RT-PCR cannot exclude detection of viral RNA in blood vessels within samples. While particular care was taken to avoid contamination by employing sterile instruments and disposable microtome blades when sampling FFPE sections for RT-PCR analyses, the main strength of our study was the complementary use of immunoperoxidase and immunofluorescent staining with different antibodies to detect viral antigen as an indicator of viral tropism. This is further strengthened by the strong concordance between these assays in our cohort, quantified by a statistically significant positive correlation between RT-PCR cycle threshold and IHC positivity (*r* = 0.87, *p* < 0.0001).

In conclusion, further investigation is required to determine the direct effects of viral invasion within the CNS, with particular regard to cases of long-lasting infection and in COVID-19 survivors.

## Methods

Hospitalized patients who died following a diagnosis of SARS-CoV-2 infection in the Veneto Region, Italy, during the peak incidence of COVID-19 (from March 2020 to May 2021) were autopsied according to established COVID-19 infection security protocols. Inclusion criteria for the study were: a) diagnosis of SARS-CoV-2 infection confirmed by molecular testing of rhino-pharyngeal swabs and b) high-quality brain tissue samples available for histopathological and immunohistochemical analysis. Tissue quality was determined by Post-Mortem Interval (PMI) ≤ 5 days, absence of tissue maceration, fixation time ≤3 weeks and adequate formalin penetration within the tissue. A total of 24 COVID-19 patients were included in the study.

Eighteen age- and sex-matched subjects with comparable general medical conditions, predating the COVID-19 pandemic in Italy, were included as controls.

### Clinical information

Available clinical data for COVID-19 subjects and controls were examined, including ante-mortem medical history, neurological and neuroradiological findings, hospitalization time, ICU and oxygen therapy status, and prescribed medication. However, as most subjects died during the sanitary emergency of the first wave of the COVID-19 pandemic in Italy, ante-mortem clinical data were at times limited, especially when concerning post-hospitalization neurological status. This represents one of the main limitations of our study, determining significant constrains to the association between ante-mortem neurological findings and encountered neuropathological alterations, which is often not unequivocal.

### Sampling and fixation procedures

Sampled brains were immersion fixed in 4% phosphate-buffered formalin solution following autopsy (mean PMI: 3 days; range 0–5 days; average fixation time: 2–3 weeks) and subsequently sectioned for histopathological and immunohistochemical analysis. Samples of the cerebral cortex, basal ganglia, hippocampus, cerebellar cortex, deep cerebellar nuclei, choroid plexuses and meninges were obtained, while the brainstem was isolated at the level of the rostral extremity of the midbrain and extensively sampled in its whole cranio-caudal extent. The 12 cranial nerves, where available, including the olfactory bulb, tract and bifurcation, were also sampled. To preserve antigen quality, a slow dehydration and clearing protocol was performed prior to paraffin embedding (24 h mean tissue processing time).

### Histochemical and immunoperoxidase staining

Haematoxylin and Eosin staining was employed for routine histopathological evaluation. Immunoperoxidase staining was performed on a Dako EnVision Autostainer (Dako Denmark A/S, Glostrup, Denmark) according to manufacturer recommendations. Antibodies for CD3 (Polyclonal Rabbit Anti-Human, Citrate Buffer HIER, dilution 1:200, Dako Omnis, Code Number: GA503), CD20 (Monoclonal Mouse Anti-Human, Citrate Buffer HIER, dilution 1:200 Clone KP1, Dako Omnis, Code Number: M0814) and CD68 (Monoclonal Mouse Anti-Human, EDTA Buffer HIER, IHC dilution 1:5000, IF dilution 1:500, Clone L26, Dako Omnis, Code Number: M0756) were employed to characterize lympho-monocytic infiltrations. Microglial Activation was assessed using both CD68 (as above), HLA-DR Antibody (Monoclonal Rabbit Anti-Human, Citrate Buffer HIER, dilution 1:50 Clone: LN-3, Invitrogen, Thermo Fisher Scientific, Waltham, MA, USA), TMEM119 (Rabbit Anti-Human, Citrate Buffer HIER, dilution 1:250, Abcam, Code Number: ab185333), while microglial proliferation was assessed using anti-Ki-67 immunohistochemistry (Mouse Anti-Human, EDTA Buffer HIER, dilution 1:200, Spring Bioscience, Code number: M3060). Anti-GFAP immunohistochemistry (Polyclonal Rabbit Anti-Human, Proteinase K enzymatic antigen retrieval, dilution 1:1000, DAKO Omnis, Code Number: GA524) was employed to assess reactive astrogliosis. Anti-CD61 immunohistochemistry (Monoclonal Mouse Anti-Human, Citrate Buffer HIER, dilution 1:75, Clone Y2/51, Dako Omnis, Code Number: M0753) was also employed to evaluate the presence of platelet-enriched microthrombi.

Anti-SARS-CoV-2 nucleocapsid (Rabbit Anti-Human, Citrate Buffer HIER, dilution 1:7000, Sino Biologicals, 40143-R001) and -Spike Subunit 1 Antibody (Monoclonal Rabbit Anti-Human, Citrate Buffer HIER, dilution 1:100, Clone 007, Sino Biological, Code Number: 40150-R007) immunostainings were employed to evaluate viral antigens within the tissue. The expression of ACE2 Receptor protein (Rabbit Anti-Human Polyclonal, Citrate Buffer HIER, dilution 1:2000, Abcam, Code Number: ab15348) and TMPRSS-2 protein (Rabbit Anti-Human Monoclonal, Citrate Buffer HIER, dilution 1:2500, Abcam, Code Number: ab242384) was assessed within the brainstem and cerebellum, and in all sections with positive findings for viral proteins. Anti-nucleocapsid and anti-spike antibodies were validated through SARS-CoV-2 infected Vero E6 cells and autopsy-derived lung tissue from SARS-CoV-2 infected patients as positive controls; non-infected cells and lung sections deriving from autopsy cases predating COVID-19 pandemic (2017) were used as negative controls (Supplementary Fig. 1). Peroxidase reactions were repeated at least three times to ensure reaction consistency.

### Immunofluorescent staining and confocal microscopy

Fluorescent immunohistochemistry was performed manually. Antigen retrieval was performed on de-paraffinized tissue sections using Dako EnVision PTLink station according to manufacturer recommendations. Following antigen retrieval, autofluorescence was quenched with a 50 mM NH_4_Cl solution for 10 min. Sections were treated with permeabilization and blocking solution (15% vol/vol Goat Serum, 2% wt/vol BSA, 0.25% wt/vol gelatin, 0.2% wt/vol glycine in PBS) containing 0.5% Triton X-100 for 90 min before primary antibody incubation. The following antibodies were employed: CD68 (#M0756; 1:500); TMEM119 (#ab185333; 1:200); Ki-67 (#M3060; 1:200); β-III Tubulin (#T8578; 1:300); Tyrosine Hydroxylase (#T2928; 1:6000); SARS-CoV-2 Nucleocapsid Protein (#40143-R001; 1:3000); SARS-CoV-2 Spike Subunit 1 Protein (#40150-R007; 1:100); ACE2 Receptor Protein (#ab15348; 1:500) and TMPRSS-2 (#ab242384; 1:1000). Primary antibodies were diluted in blocking solution and incubated at 4 °C overnight. Alexa-Fluor plus 488 Goat anti-Mouse secondary antibody (Code number: A32723) and Alexa-Fluor plus 568 anti-Rabbit secondary antibody (Code number: A-11011) were diluted 1:200 in blocking solution as above and incubated for 60 min at room temperature. To further avoid background signal and tissue autofluorescence, slides were incubated for 10 min in 0.5% Sudan Black B solution in 70% ethanol at room temperature and abundantly washed with PBS, followed by Hoechst 33258 nuclear staining (Invitrogen, dilution: 1:10,000 in PBS) for 10 min. Slides were mounted and coverslipped with Mowiol solution (prepared with Mowiol 4–88 reagent, MerckMillipore, Code number: 475904-100GM). Confocal immunofluorescence z-stack images were acquired on a Leica SP5 Laser Scanning Confocal Microscope using a HC PL FLUOTAR × 20/0.50 Dry or HCX PL APO lambda blue ×40/1.40 Oil objectives. Images were acquired at a 16-bit intensity resolution over 2048 × 2048 pixels. Z-stacks images were converted into digital maximum intensity z-projections, processed, and analyzed using ImageJ software, as previously reported^46–48^.

### RT-PCR analyses

Viral RNA analysis was performed on 20 µm thick paraffin-embedded sections collected in sterile 2 ml Eppendorf vials; disposable microtome blades and tongs were changed for each section to reduce contamination risk. Real-time RT-PCR analyses were performed to detect SARS-CoV-2 genome sequences. Briefly, total RNA was purified from selected material using a RecoverAll™ Total Nucleic Acid Isolation kit (Thermo Fisher Scientific) following the manufacturer’s instructions. One-step real-time RT-PCR assays targeting SARS-CoV-2 nucleocapsid (N) coding region and subgenomic RNA were run on ABI 7900HT Sequence Detection Systems (Thermo Fisher Scientific), as previously reported^27^.

### Histopathological and morphometrical evaluation

Slides were examined by three independent histopathologists and morphologists blind to patient clinical findings and COVID-19 status. Disagreements were resolved by consensus. The degree of brainstem hypoxic/ischemic damage, astrogliosis and microgliosis were classified using a four-tiered semi-quantitative approach for each evaluated section, while microglial density and activation was assessed by the means of digitally-assisted immunoreactivity quantification by three independent evaluators.

### Quantification of activated microglia

The degree of microgliosis was assessed through a digitally-assisted quantification approach at the level of the medulla, pons and mesencephalon. For each subject, standard sections passing through the area postrema (medulla), locus coeruleus (pons) and decussation of the superior cerebellar peduncles or red nucleus (midbrain) underwent TMEM119 immunoperoxidase staining and TMEM119/CD68 double fluorescent immunohistochemistry. TMEM119+ structures with visible nucleus and microglial-compatible morphology were classified as microglial cells, while TMEM119−/CD68+ elements with compatible morphology were classified as monocyte/macrophages. Ramifications and cell processes without a visible nucleus were excluded from our analysis in order to avoid overestimation of cell densities by including neighboring structures belonging to adjacent sections. Morphometrical evaluation occurred within six counting fields (fields of view, FOV) spanning across the dorsal-to-ventral axis of the sections; FOV boundaries and anatomical landmarks are summarized in Supplementary Table 1 for each level of sectioning. The number of immunoreactivities per mm^2^ was calculated for each counting field and assigned to one anatomical compartment (i.e., tegmentum, tectum and basis), based on their topography according to Mai and Paxinos^49^. Comparisons and statistical evaluations were conducted per individual counting field, anatomical compartment and level of section (medulla, pons, midbrain). To assess the degree of lysosomal-activity as a marker for microglia phagocytic activity, CD68 immunoreactive area (expressed as percentage of CD68+ immunoreactive area within a counting field, or A%) for five randomly selected counting fields at each level of sectioning was computed through particle analysis of the green fluorescent channel on ImageJ software.

### Statistical analyses

Statistical analyses and visualizations were performed using GraphPad Prism 9. Microglial densities (microglia/mm^2^) between individual counting fields (FOVs) in COVID-19 subjects (Figs. 8d, 9d and 10d), as well as differences between anatomical compartments in COVID-19 subgroups (Figs. 8h and 10g) and in COVID-19 versus controls (Figs. 6e, 8f, 9e and 10e) were determined by Welch one-way ANOVA tests corrected for Dunnett’s multiple comparisons. Differences in microglial densities within subgroups of the COVID-19 cohort in Figs. 8f, 9e and 10e were analyzed by *t-*tests with Welch’s correction. Correlation matrices in Fig. 8e were computed as Spearman’s rho for continuous variables and as point-biserial correlations for nominal—continuous variables. Spearman’s rho and linear regression was performed in Fig. 5f. Further statistical details for each plot can be found in the corresponding figure legend. Throughout the manuscript * indicates *p* < 0.05, **p < 0.01, ***p < 0.001 and ****p < 0.0001*.*

### Ethical approval

All procedures were carried out in accordance to the Declaration of Helsinki. Samples were anonymous to the investigators and used in accordance with the directives of the Committee of the Ministers of EU member states on the use of samples of human origin for research. Donor subjects gave their written informed consent prior to death according to regulations of the Body Donation Program of the Institute of Human Anatomy, Department of Neuroscience, University of Padova.

### Reporting summary

Further information on research design is available in the Nature Research Reporting Summary linked to this article.

## Supplementary information

Supplementary Material

Reporting Summary

## Data Availability

All data and source measurements are available by the corresponding author upon request.
