# Supplementary material for: Detection of SARS-CoV-2 viral proteins and genomic sequences in human brainstem nuclei
Source: NPJ Parkinsons Dis. 2023 Feb 13;9:25. doi: 10.1038/s41531-023-00467-3 (PMC9924897; doi:10.1038/s41531-023-00467-3)
Supplement: Supplementary file 1 — Supplementary Material [file 41531_2023_467_MOESM1_ESM.pdf]

Supplementary Material

Aron Emmi, Stefania Rizzo, Luisa Barzon, Michele Sandre, Elisa Carturan, Alessandro Sinigaglia, Silvia Riccetti, Mila della Barbera, Rafael Boscolo-Berto, Patrizia Cocco, Veronica Macchi, Angelo Antonini, Monica De Gaspari, Cristina Basso, Raffaele De Caro*, Andrea Porzionato

SUPPLEMENTARY TABLE 1. Anatomical boundaries and main structures contained in each examined Field of View, divided per level of sectioning.

LEVEL OF SECTIONING	**FOV1**	**FOV2**	**FOV3**	**FOV4**	**FOV5**	**FOV6**
MEDULLA	Tegmentum: Dorsal Motor Nucleus of the Vagus, Solitary Tract Nucleus, Area Postrema	Tegmentum: Hypoglossal Nucleus, Intercalate Nucleus, Medial Longitudinal Lemniscus, Dorsal Medullary Reticular Formation	Tegmentum: Nucleus Ambiguus, Ventral Medullary Reticular Formation.	Basis: Medial Lemniscus, Medial Accessory Olivary Nucleus, Inferior Olivary Complex (Medial Part)	Basis: Inferior Olivary Complex (Lateral Part).	Basis: Pyramidal Tract.
PONS	Tegmentum: Dorsal Tegmentum, Locus Coeruleus, Medial Longitudinal Fasciculus.	Tegmentum: Ventrolateral Tegmentum, Spinal Trigeminal Tract.	Tegmentum: Ventromedial Tegmentum, Medial lemniscus, Central Tegmental Tract.	Basis: Dorsomedial basis pontis, basilary nuclei of the pons, cortico-spinal tract.	Basis: Lateral basis pontis, basilary nuclei of the pons, cortico-spinal tract.	Basis: Ventromedial basis pontis, basilary nuclei of the pons, cortico-spinal tract.
MIDBRAIN	Tegmentum: Lateral part of the Substantia Nigra, Red Nucleus	Tegmentum: Medial part of the Substantia Nigra.	Tectum: Quadrigeminal Plate, Periacqueductal Gray.	Tegmentum / Tectum interface, Mesencephalic reticular formation.	Basis: lateral part of the cerebral peduncle.	Basis: medial part of the cerebral peduncle.

SUPPLEMENTARY TABLE 2. Histopathological and Immunohistochemical findings in the COVID-19 Subjects. N and S indicate the viral antigen detected in the tissue. N, Nucleocapsid Protein; S, Spike Protein; RT-CT, Real Time Cycle.

	Mild
	Moderate
	Severe
	Absent / Negative
	Present / Positive

CASE	SAMPLE / LEVEL	ASTROGLIOSIS (GFAP)	MICROGLIOSIS (TMEM119-CD68-HLA-DR)	THROMBOSES / SMALL VESSEL THROMBOSES (H&E – CD61)	VIRAL PROTEINS (IHC)	VIRAL RNA (RT-PCR)
#1	Medulla Oblongata					
	Pons					
	Midbrain					
	Cerebellar Cortex					
	Basal Ganglia					
	Frontal Cortex					
	Olfactory bulbs and tracts					
	Cranial Nerves (III-XI)					

	Leptomeninges					
	Choroid Plexuses					
**#2**	Medulla Oblongata					
	Pons					
	Midbrain					
	Cerebellar Cortex					
	Basal Ganglia					
	Frontal Cortex					
	Olfactory bulbs and tracts					
	Cranial Nerves (III-XI)					
	Leptomeninges				**S**	
	Choroid Plexuses					
**#3**	Medulla Oblongata				**S, N**	
	Pons				**S, N**	
	Midbrain				**S, N**	
	Cerebellar cortex					
	Basal Ganglia				**S**	

	Frontal Cortex					
	Olfactory bulbs and tracts					
	Cranial Nerves (III-XI)					
	Leptomeninges				S	
	Choroid Plexuses					
**#4**	Medulla Oblongata					
	Pons					
	Midbrain					
	Cerebellar Cortex					
	Basal Ganglia					
	Frontal Cortex					
	Olfactory bulbs and tracts					
	Leptomeninges					
	Choroid Plexuses					
**#5**	Medulla Oblongata					
	Pons					

	Midbrain					
	Cerebellar Cortex					
	Deep cerebellar nuclei					
	Basal Ganglia					
	Hippocampus					
	Frontal Cortex					
	Olfactory bulbs and tracts					
	Leptomeninges					
	Choroid Plexuses					
**#6**	Medulla Oblongata					
	Pons					
	Midbrain					
	Cerebellar Cortex					
	Deep cerebellar nuclei					
	Basal Ganglia					
	Hippocampus					

	Frontal Cortex					
	Leptomeninges					
	Choroid Plexuses					
**#7**	Medulla Oblongata				**S**	RT-CT=35,46
	Pons					
	Midbrain				**S**	RT-CT=39,96
	Cerebellar Cortex					
	Deep cerebellar nuclei					
	Basal Ganglia					
	Hippocampus				**S, N**	RT-CT=38,04
	Frontal Cortex					
	Leptomeninges					
	Choroid Plexuses					
**#8**	Medulla Oblongata					
	Pons					
	Midbrain					

	Cerebellar Cortex					
	Deep cerebellar nuclei					
	Basal Ganglia					
	Hippocampus					
	Frontal Cortex					
	Leptomeninges					
	Choroid Plexuses					
**#9**	Medulla Oblongata				**S, N**	RT-CT=37,4
	Pons					
	Midbrain				**N**	RT-CT=38,14
	Cerebellar Cortex					
	Deep cerebellar nuclei					
	Basal Ganglia					
	Hippocampus					
	Frontal Cortex					
	Leptomeninges				**S**	RT-CT=37,4
	Choroid Plexuses					

**#10**	Medulla Oblongata				**N**	ND
	Pons					
	Midbrain				**N**	ND
	Cerebellar Cortex					
	Deep cerebellar nuclei					
	Basal Ganglia					
	Frontal Cortex and subcortex				**S, N**	RT-CT=37,61
	Leptomeninges					
	Choroid Plexuses					
**#11**	Medulla Oblongata					
	Pons					
	Midbrain					
	Cerebellar Cortex					
	Deep cerebellar nuclei					
	Basal Ganglia					

	Frontal Cortex					
	Parietal Cortex				S	RT-CT=38,00
	Leptomeninges					
	Choroid Plexuses					
**#12**	Medulla Oblongata					
	Pons					
	Midbrain					
	Cerebellar Cortex					
	Deep cerebellar nuclei					
	Basal Ganglia					
	Frontal Cortex					
	Leptomeninges					
	Choroid Plexuses					
**#13**	Medulla Oblongata					
	Pons					
	Midbrain					

	Cerebellar Cortex					
	Deep cerebellar nuclei					
	Basal Ganglia					
	Frontal Cortex					
	Leptomeninges					
	Choroid Plexuses					
**#14**	Medulla Oblongata					
	Pons					
	Midbrain					
	Cerebellar Cortex					
	Deep cerebellar nuclei					
	Basal Ganglia					
	Frontal Cortex					
	Leptomeninges					
	Choroid Plexuses					
**#15* Poor tissue quality**	Medulla Oblongata					
	Pons					

	Midbrain					
	Deep cerebellar nuclei					
	Basal Ganglia					
	Frontal Cortex					
	Leptomeninges					
	Choroid Plexuses					
**#16**	Medulla Oblongata					
	Pons					
	Midbrain					
	Cerebellar Cortex					
	Deep cerebellar nuclei					
	Basal Ganglia					RT-CT=37,47
	Frontal Cortex					
	Leptomeninges					
	Choroid Plexuses					
**#17**	Medulla Oblongata				**S**	RT-CT=38,34

	Pons					
	Midbrain				**N**	ND
	Cerebellar Cortex					
	Deep cerebellar nuclei				**S, N**	ND
	Basal Ganglia					
	Frontal Cortex					
	Leptomeninges					
	Choroid Plexuses					
**#18**	Medulla Oblongata					
	Pons					
	Midbrain					
	Cerebellar Cortex					
	Deep cerebellar nuclei				**S**	RT-CT=37,33
	Basal Ganglia					
	Frontal Cortex					
	Leptomeninges					
	Choroid Plexuses					

**#19**	Pons					
	Midbrain					
	Cerebellar Cortex					
	Deep cerebellar nuclei					
	Basal Ganglia					
	Occipital Cortex					
	Frontal Cortex					
	Parietal Cortex					
	Temporal Cortex					
**#20**	Pons					
	Midbrain					
	Cerebellar Cortex					
	Deep cerebellar nuclei					
	Basal Ganglia					
	Occipital Cortex					
	Frontal Cortex					

	Parietal Cortex					
	Temporal Cortex					
**#21**	Medulla Oblongata					
	Pons					
	Midbrain					
	Cerebellar Cortex					
	Deep cerebellar nuclei					
	Basal Ganglia					
	Hippocampus					
	Frontal Cortex					
	Parietal Cortex					
	Temporal Cortex					
	Leptomeninges					
	Choroid Plexuses					
**#22**	Medulla Oblongata					
	Pons					
	Midbrain					

	Cerebellar Cortex					
	Deep cerebellar nuclei					
	Basal Ganglia					
	Hippocampus					
	Frontal Cortex					
	Parietal Cortex					
	Temporal Cortex					
	Leptomeninges					
	Choroid Plexuses					
**#23**	Medulla Oblongata					
	Pons					
	Midbrain					
	Cerebellar Cortex					
	Deep cerebellar nuclei					
	Basal Ganglia					
	Hippocampus					

	Frontal Cortex					
	Parietal Cortex					
	Temporal Cortex					
	Leptomeninges					
	Choroid Plexuses					
**#24**	Medulla Oblongata					
	Pons					
	Midbrain					
	Cerebellar Cortex					
	Deep cerebellar nuclei					
	Basal Ganglia					
	Hippocampus					
	Frontal Cortex					
	Parietal Cortex					
	Temporal Cortex					
	Leptomeninges					
	Choroid Plexuses					

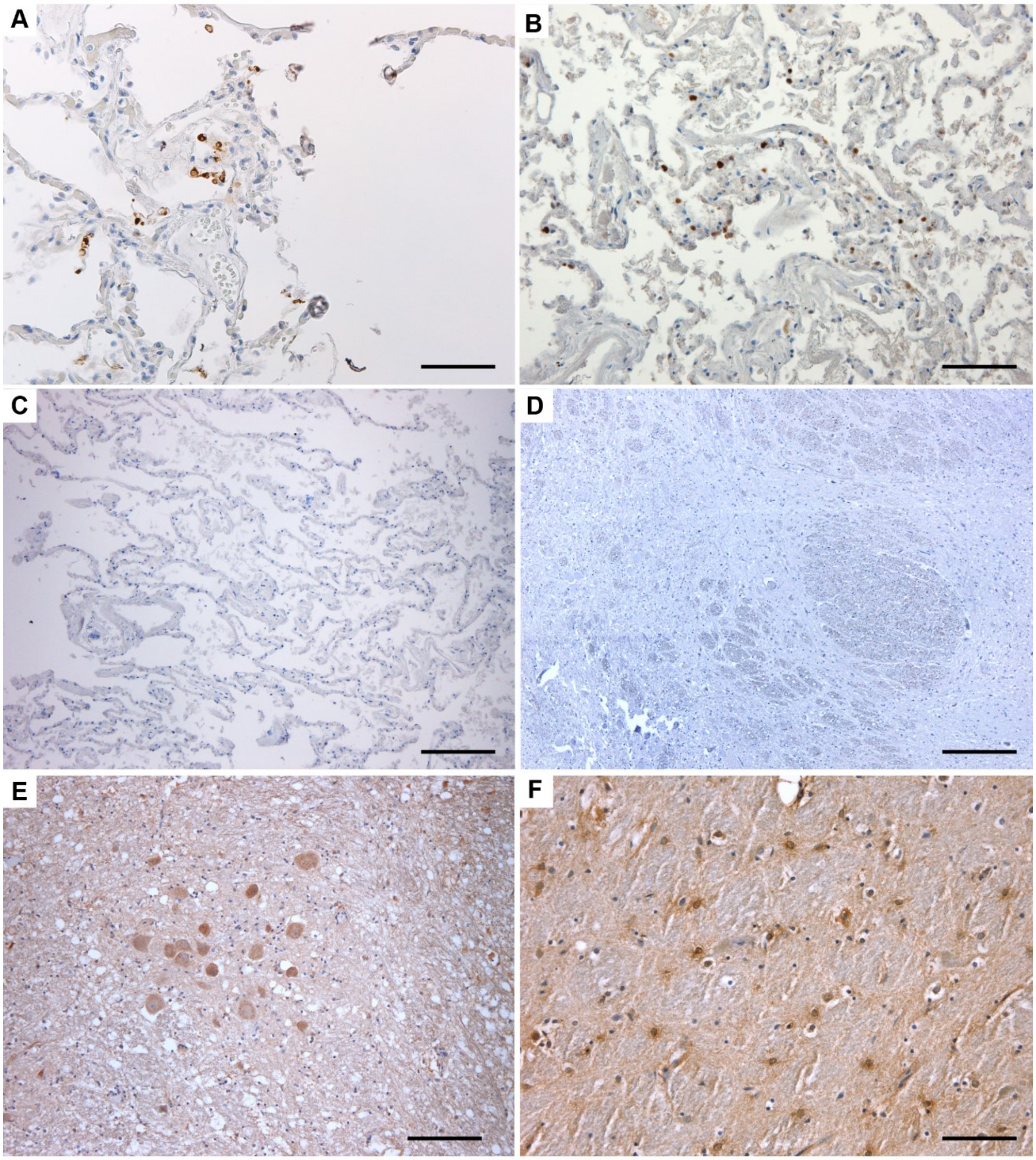

Supplementary Figure 1. A) SARS-CoV-2 Spike Protein immunohistochemistry on RT-PCR+ lung tissue. **B)** S SARS-CoV-2 Nucleocapsid Protein immunohistochemistry on RT-PCR+ lung tissue. **C)** SARS-CoV-2 Spike Protein immunohistochemistry on lung tissue from control subjects, showing no immunoreactive elements. **D)** SARS-CoV-2 Spike Protein immunohistochemistry on brainstem tissue (solitary tract nucleus) from control subjects, showing no immunoreactive cells. **E)** ACE-2 Receptor immunohistochemistry at the level of the nucleus ambiguus in the medulla oblongata of a COVID-19 subject, showing diffuse immunoreactivity in the soma and surrounding neuropilum. **F)** TMPRSS-2 protein immunoreactivity in the mesencephalic tegmentum showing moderate reactivity of non-neuronal cells. *Scale Bars: 200μm (C,D); 100μm (A,B,E,F).*

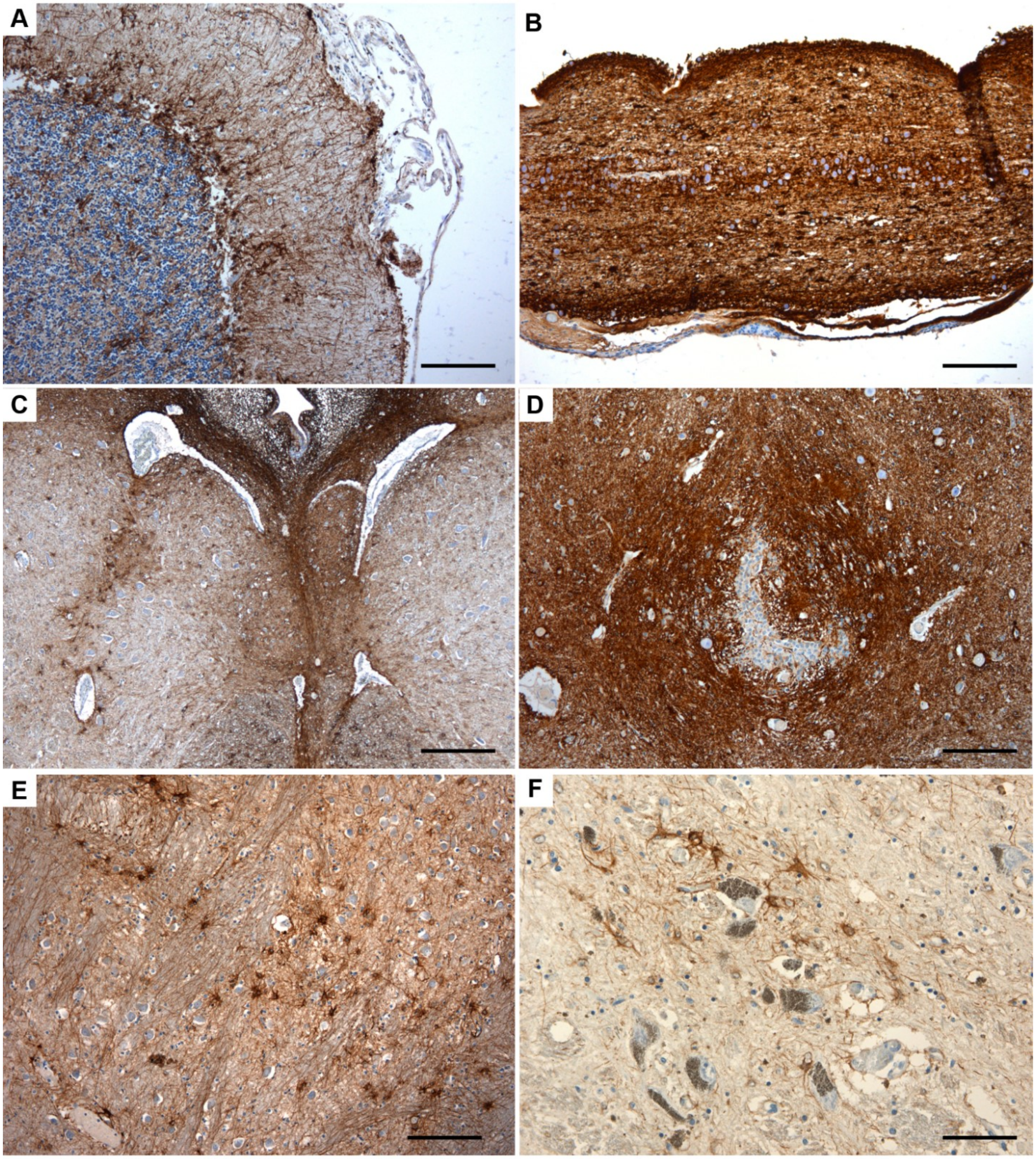

Supplementary Figure 2. GFAP Immunohistochemistry. **A)** Reactive Bergmann Glia at the level of the cerebellar cortex in Subject #2. **B)** Marked astrogliosis with numerous corpora amylacea could be appreciated in this sagittal section of the olfactory tract. **C)** Reactive astrocytosis was marked within the subependymal regions of the medullary tegmentum, also displaying numerous reactive astrocytes within the hypoglossal nucleus. **D)** Reactive Astrocytosis surrounding the central canal of the lower medulla. **E)** Reactive astrocytes within the basilary pons in Subject #3. **F)** Reactive astrocytes at the level of the substantia nigra in the midbrain. *Scale Bars: 200µm (C); 100µm (A,B,D,E); 50µm (F).*

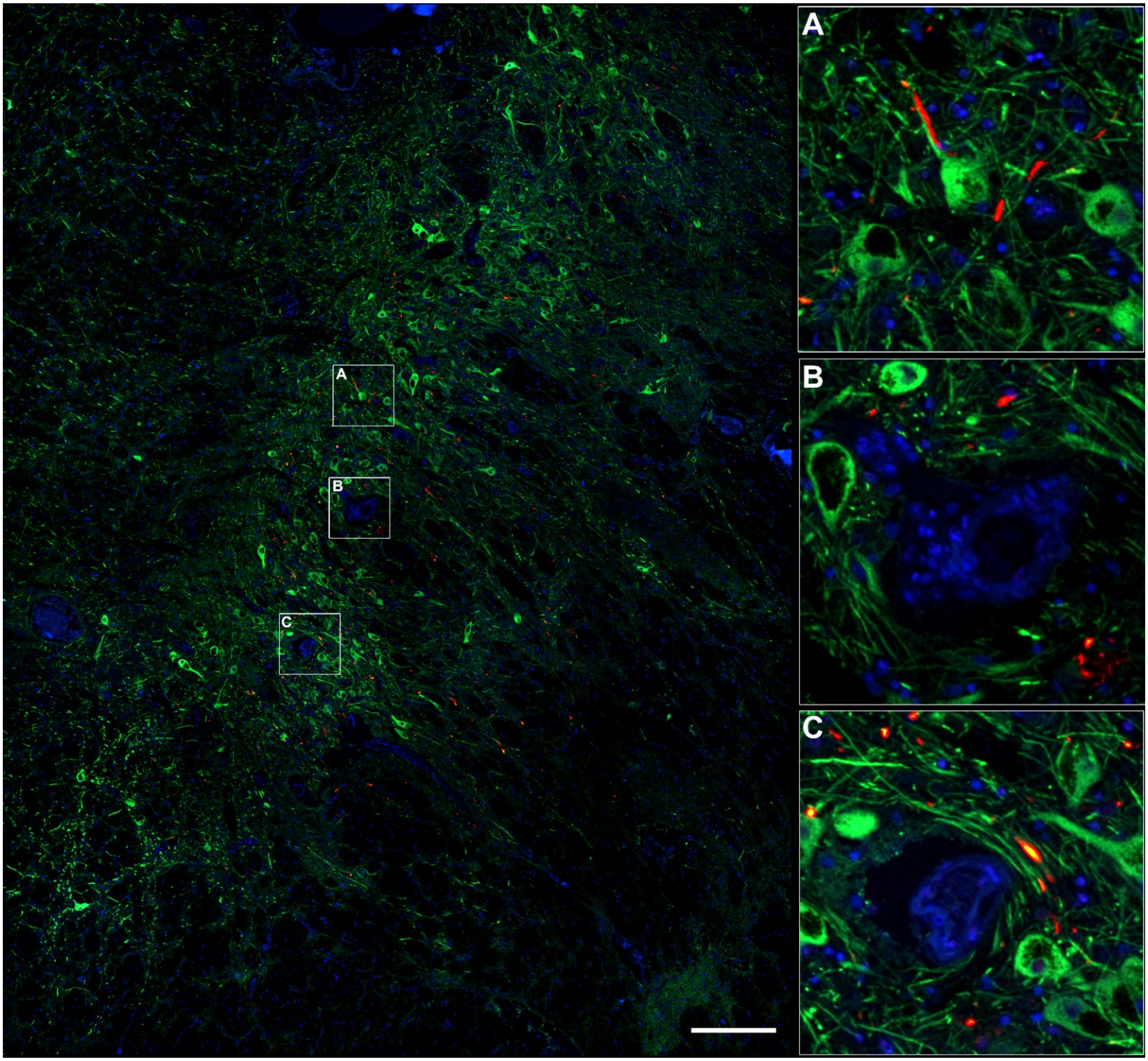

Supplementary Figure 3. Tyrosine Hydroxylase (green), Nucleocapsid Protein (red) and Hoechst (blue) immunofluorescent staining. Nucleocapsid protein immunoreactivity can be detected within the boundaries of the substantia nigra in the midbrain and may be found within tyrosine hydroxylase positive neurites and neuronal somata.
