# Supplementary material for: Detection of SARS-CoV-2 viral proteins and genomic sequences in human brainstem nuclei
Source: NPJ Parkinsons Dis. 2023 Feb 13;9:25. doi: 10.1038/s41531-023-00467-3 (PMC9924897; doi:10.1038/s41531-023-00467-3)
Supplement: Supplementary file 2 — Reporting Summary [file 41531_2023_467_MOESM2_ESM.pdf]

nature portfolio

Corresponding author(s): Prof. Raffaele De Caro - Prof. Angelo Antonini

Last updated by author(s): Jan 3, 2023

Reporting Summary

Nature Portfolio wishes to improve the reproducibility of the work that we publish. This form provides structure for consistency and transparency in reporting. For further information on Nature Portfolio policies, see our Editorial Policies and the Editorial Policy Checklist.

Statistics

For all statistical analyses, confirm that the following items are present in the figure legend, table legend, main text, or Methods section.

n/a	Confirmed	
☐	☒	The exact sample size (*n*) for each experimental group/condition, given as a discrete number and unit of measurement
☐	☒	A statement on whether measurements were taken from distinct samples or whether the same sample was measured repeatedly
☐	☒	The statistical test(s) used AND whether they are one- or two-sided<br>*Only common tests should be described solely by name; describe more complex techniques in the Methods section.*
☐	☒	A description of all covariates tested
☐	☒	A description of any assumptions or corrections, such as tests of normality and adjustment for multiple comparisons
☐	☒	A full description of the statistical parameters including central tendency (e.g. means) or other basic estimates (e.g. regression coefficient) AND variation (e.g. standard deviation) or associated estimates of uncertainty (e.g. confidence intervals)
☐	☒	For null hypothesis testing, the test statistic (e.g. *F*, *t*, *r*) with confidence intervals, effect sizes, degrees of freedom and *P* value noted<br>*Give P values as exact values whenever suitable.*
☒	☐	For Bayesian analysis, information on the choice of priors and Markov chain Monte Carlo settings
☒	☐	For hierarchical and complex designs, identification of the appropriate level for tests and full reporting of outcomes
☐	☒	Estimates of effect sizes (e.g. Cohen's *d*, Pearson's *r*), indicating how they were calculated

Our web collection on statistics for biologists contains articles on many of the points above.

Software and code

Policy information about availability of computer code

Data collection	N/A
Data analysis	N/A

For manuscripts utilizing custom algorithms or software that are central to the research but not yet described in published literature, software must be made available to editors and reviewers. We strongly encourage code deposition in a community repository (e.g. GitHub). See the Nature Portfolio guidelines for submitting code & software for further information.

Data

Policy information about availability of data

All manuscripts must include a data availability statement. This statement should provide the following information, where applicable:

- Accession codes, unique identifiers, or web links for publicly available datasets
- A description of any restrictions on data availability
- For clinical datasets or third party data, please ensure that the statement adheres to our policy

Data is available from the corresponding author upon request; raw data underlying graphs and statistical analyses has been provided to the editor, reviewers, and is available upon reasonable request.

Field-specific reporting

Please select the one below that is the best fit for your research. If you are not sure, read the appropriate sections before making your selection.

☒ Life sciences ☐ Behavioural & social sciences ☐ Ecological, evolutionary & environmental sciences

For a reference copy of the document with all sections, see nature.com/documents/nr-reporting-summary-flat.pdf

Life sciences study design

All studies must disclose on these points even when the disclosure is negative.

Sample size	For the COVID-19 group, sample size was based upon 1) availability of autopsy brain tissue of hospitalized subjects with positive RT-PCR testing for SARS-CoV-2 and 2) quality of brain tissues. 24 patients were selected based upon these criteria. For the control group, 18 age-and sex- matched subjects with pneumonia / respiratory failure as main cause of death were selected.
Data exclusions	Immunoperoxidase and Immunofluorescent staining was not performed on one subject of the COVID-19 group (#15) due to issues with tissue processing during antigen retrieval; however, routine gross and histopathological examination through haematoxylin&eosin staining was performed.<br>Concerning the quantification of activated microglia, estimation of cell density was not performed two subjects (#20-#21) at the level of the medulla due to incomplete sectioning of the brainstem upon autopsy.<br>In-situ hybridization and immunogold TEM was also performed for some of the IHC+ samples, but results were not satisfactory due to prolonged fixation in formalin and paraffin embedding of the specimens (data not shown).
Replication	Immunoperoxidase and immunofluorescent staining procedures were repeated at least three times to ensure reaction consistency. All reported immunohistochemical staining data was replicated by two independent histotechnologists blind to clinical data.
Randomization	Not relevant for this study, due to involvement of human autopsy tissue.
Blinding	Histopathological evaluation was performed by three independent morphologists and neuropathologists blind to patient clinical data. Disagreements were resolved by consensus. Morphometrical analyses (quantification of reactive microglia) was performed blind to both subject group and patient clinical data.

Reporting for specific materials, systems and methods

We require information from authors about some types of materials, experimental systems and methods used in many studies. Here, indicate whether each material, system or method listed is relevant to your study. If you are not sure if a list item applies to your research, read the appropriate section before selecting a response.

Materials & experimental systems

n/a	Involved in the study	
☐	☒	Antibodies
☒	☐	Eukaryotic cell lines
☒	☐	Palaeontology and archaeology
☒	☐	Animals and other organisms
☐	☒	Human research participants
☒	☐	Clinical data
☒	☐	Dual use research of concern

Methods

n/a	Involved in the study	
☒	☐	ChIP-seq
☒	☐	Flow cytometry
☒	☐	MRI-based neuroimaging

Antibodies

Antibodies used	Immunohistochemistry Antibodies: CD3 (Polyclonal Rabbit Anti-Human, Citrate Buffer HIER, dilution 1:200, Dako Omnis, Code Number: GA503), CD20 (Monoclonal Mouse Anti-Human, Citrate Buffer HIER, dilution 1:200 Clone KP1, Dako Omnis, Code Number: M0814) CD68 (Monoclonal Mouse Anti-Human, EDTA Buffer HIER, IHC dilution 1:5000, IF dilution 1:500, Clone L26, Dako Omnis, Code Number: M0756), HLA-DR (Monoclonal Rabbit Anti-Human, Citrate Buffer HIER, dilution 1:50 Clone: LN-3, Invitrogen, Thermo Fisher Scientific, Waltham, MA, USA), TMEM119 (Rabbit Anti-Human, Citrate Buffer HIER, dilution 1:250, Abcam, Code Number: ab185333), Ki-67 (Mouse Anti-Human, EDTA Buffer HIER, dilution 1:200, Spring Bioscience, Code number: M3060), GFAP (Polyclonal Rabbit Anti-Human, Proteinase K enzymatic antigen retrieval, dilution 1:1000, DAKO Omnis, Code Number: GA524), CD61 (Monoclonal Mouse Anti-Human, Citrate Buffer HIER, dilution 1:75, Clone Y2/51, Dako Omnis, Code Number: M0753), SARS-CoV-2 Nucleocapsid (Rabbit Anti-Human, Citrate Buffer HIER, dilution 1:7000, Sino Biologicals, 40143-R001), SARS-CoV-2 Spike Subunit 1 Antibody (Monoclonal Rabbit Anti-Human, Citrate Buffer HIER, dilution 1:100, Clone 007, Sino Biological, Code Number: 40150-R007), ACE2 Receptor (Rabbit Anti-Human Polyclonal, Citrate Buffer HIER, dilution 1:2000, Abcam, Code Number: ab15348), TMPRSS-2 protein (Rabbit Anti-Human Monoclonal, Citrate Buffer HIER, dilution 1:2500, Abcam, Code Number: ab242384), β-III Tubulin (#T8578; dilution 1:300), Tyrosine Hydroxylase (#T2928; dilution 1:6000).

Validation	SARS-CoV-2 Nucleocapsid and Spike Subunit 1 Antibodies were validated through SARS-CoV-2 infected Vero E6 cells and autopsy-derived lung tissue from SARS-CoV-2 infected patients as positive controls. Non-infected cells and lung sections deriving from autopsy cases predating COVID-19 pandemic were used as negative controls (data shown in Supplementary Figure 1).

Human research participants

Policy information about studies involving human research participants

Population characteristics	Twenty-four (24) COVID-19 patients were included in the study (11 females, 13 males). The mean age of the included subjects was 73±13.7 years. Available clinical information, hospitalization time, neurological findings, ante-mortem head CT findings and autopsy findings were reported in table 1.<br><br>Eighteen (18) sex- and age-matched control subjects were included in the study (8 female, 10 male). The mean age of included controls was 72±12 years. Available clinical information and autopsy findings were reported in table 2.
Recruitment	Not applicable for this type of study. Sample size was based upon 1) availability of autopsy brain tissue and 2) adequate quality of brain tissues.
Ethics oversight	All procedures were carried out in accordance to the Declaration of Helsinki. Samples were anonymous to the investigators and used in accordance with the directives of the Committee of the Ministers of EuropeanUnion member states on the use of samples of human origin for research.

Note that full information on the approval of the study protocol must also be provided in the manuscript.
